# Finite Element Modeling of the Combined Faradaic and Electrostatic Contributions to the Voltammetric Response of Monolayer Redox Films

**DOI:** 10.1021/acs.analchem.2c01976

**Published:** 2022-09-07

**Authors:** Katherine J. Levey, Martin A. Edwards, Henry S. White, Julie V. Macpherson

**Affiliations:** † Department of Chemistry, University of Warwick, CoventryCV4 7AL, U.K.; ‡ Centre for Diamond Science and Technology, University of Warwick, CoventryCV4 7AL, U.K.; § Department of Chemistry & Biochemistry, University of Arkansas, Fayetteville, Arkansas72701, United States; ∥ Department of Chemistry, University of Utah, 315S 1400E, Salt Lake City, Utah84112, United States

## Abstract

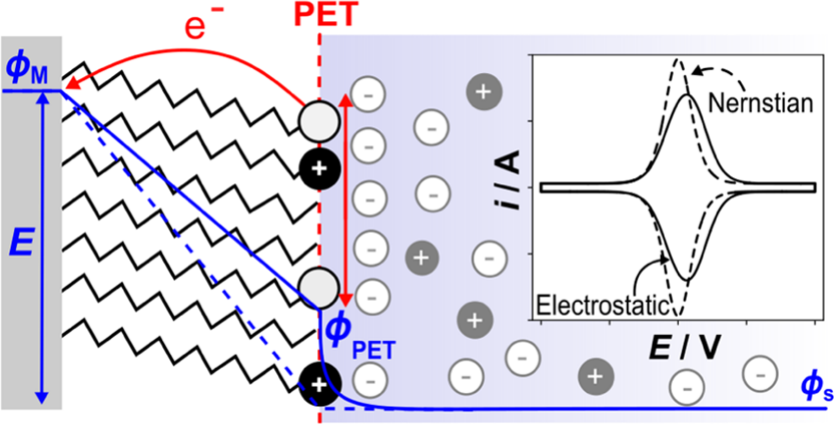

The voltammetric response of electrodes coated with a redox-active monolayer is computed by finite element simulations based on a generalized model that couples the Butler–Volmer, Nernst–Planck, and Poisson equations. This model represents the most complete treatment of the voltammetric response of a redox film to date and is made accessible to the experimentalist via the use of finite element modeling and a COMSOL-generated report. The model yields a full description of the electric potential and charge distributions across the monolayer and bulk solution, including the potential distribution associated with ohmic resistance. In this way, it is possible to properly account for electrostatic effects at the molecular film/electrolyte interface, which are present due to the changing charge states of the redox head groups as they undergo electron transfer, under both equilibrium and nonequilibrium conditions. Specifically, our numerical simulations significantly extend previous theoretical predictions by including the effects of finite electron-transfer rates (*k*^0^) and electrolyte conductivity. Distortion of the voltammetric wave due to ohmic potential drop is shown to be a function of electrolyte concentration and scan rate, in agreement with experimental observations. The commonly used Laviron analysis for the determination of *k*^0^ fails to account for ohmic drop effects, which may be non-negligible at high scan rates. This model provides a more accurate alternative for *k*^0^ determination at all scan rates. The electric potential and charge distributions across an electrochemically inactive monolayer and electrolyte solution are also simulated as a function of applied potential and are found to agree with the Gouy-Chapman-Stern theory.

## Introduction

Self-assembled monolayers (SAMs) containing a terminal redox-active moiety are a well-studied model system for probing the fundamental factors that control the rate of interfacial electron transfer, under conditions where mass transport of the redox species can be neglected.^1−7^ Such understanding is aided by the ability to vary both the distance between the redox head group and the electrode surface and the chemistry of the bridging molecules and redox head groups. Redox-active SAMs have also found use in more applied applications including electrochemical sensing^8,9^ and molecular electronic devices.^10−13^

Many of the prior experimental studies employed cyclic voltammetry (CV) to probe the electrochemical response of the redox film,^2,14,15^ where parameters such as the peak potential (*E*_p_), peak current (*i*_p_), and peak full width at half maximum (fwhm) were used in the analysis of the data.^16^ Early analytical theory derived the theoretical values for these parameters, under reversible electron transfer conditions, by combining the Nernst equation with a Langmuir adsorption description of the redox film.^17^ This theory determined symmetrical peak-shaped CV responses with an fwhm equal to 90.6/*n* mV (*n* is the number of electrons transferred) and an *E*_p_ equaling the formal redox potential (*E*^0’^). The fwhm and *E*_p_ were also predicted to be independent of the total surface coverage of the redox-active head groups (Γ_T_). However, experimentally, the observed voltammograms often showed deviations from this theory, suggesting that these early analytical descriptions did not fully capture the physical processes taking place.^18,19^

Attempts to account for the observed deviations were made by noting that the oxidized/reduced (or both) form of the redox species will introduce a charge at the molecular film/electrolyte interface, which was not accounted for in the early models. This interfacial potential distribution has also been shown to affect the voltammetric response of semiconductor electrodes modified with redox monolayers.^20^ Laviron developed a phenomenological model that used a Frumkin adsorption isotherm with an adjustable parameter to model interactions between the charged molecules in the redox film.^21−24^ In later work, Smith and White used analytical expressions to compute the interfacial potential distribution across the charged redox film/electrolyte interface in response to faradaic reduction or oxidation of the redox head groups.^25^ Further refinements by Fawcett and Andreu et al. considered the discrete nature of the charged redox head groups, as well as ion-pairing between the redox head groups and supporting electrolyte ions.^26,27^ Ohtani et al, considered ion pairing and related the phenomenological interaction parameter (developed by Laviron) to the physical properties of the film that determine the electric potential distribution across the interface, for example, thickness and dielectric constant of the molecular film.^28^ Ohtani also expanded the descriptions further by including finite electron-transfer kinetics.^29^

Whilst these models provided quantitative descriptions to account for the observed deviations in the peak fwhm and *E*^0’^, they were all limited by the assumption that the electrical double layer is at equilibrium. This becomes especially problematic when using high voltammetric scan rates and/or low supporting electrolyte concentrations. Under these conditions, the finite transport rates of supporting electrolyte ions prevent the establishment of an equilibrium double layer structure on voltammetric time scales. This results in an electric potential varying solution resistance and capacitance. Whilst Amatore et al^30^ and Feldberg^31^ attempted to account for capacitive and resistive effects on the voltammetric response of redox film electrodes, they used a circuit analysis approach and assumed potential independent values for resistance and capacitance, which is not correct under nonequilibrium conditions.

To address this problem, we present a numerical (finite element) simulation approach to describe the coupling of redox film chemistry with the mass transport of electrolyte ions in the electrolyte solution. This enables the prediction of the voltammetric response of redox-active monolayers under both equilibrium and nonequilibrium conditions. It also allows us to explore how the different physical processes, which control nonfaradaic and faradaic charge transfer, are coupled to one another and to the motion of electrolyte ions. Overall, our simulations provide the most complete description to date of the voltammetric response of a redox-active monolayer that includes the effect of the interfacial potential distribution, finite electron-transfer kinetics, and electrolyte transport.

## Model and Theory

### Molecular Redox Film

**Figure 1:**
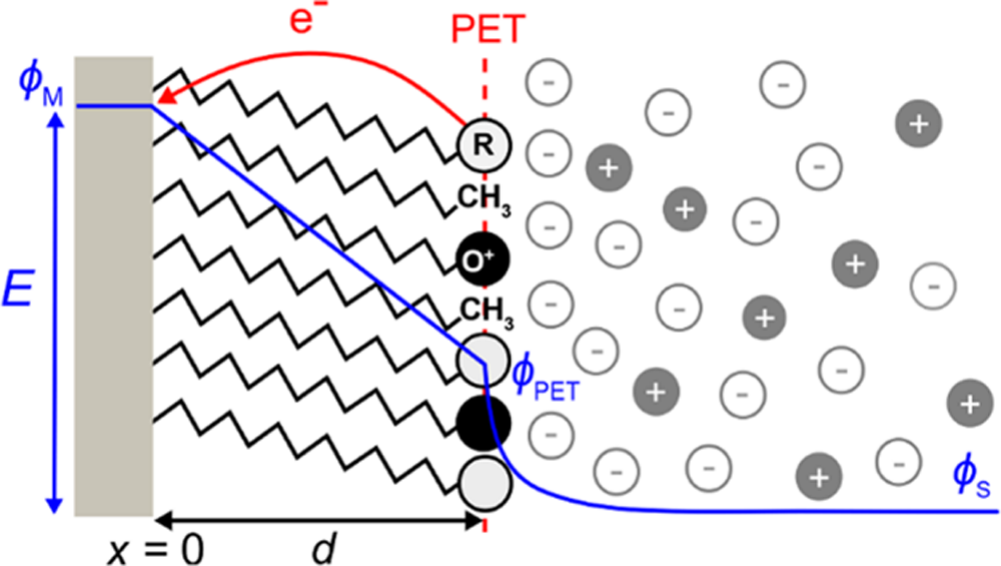
Schematic of a redox-active (O^+^/R) film on a metal electrode in contact with an electrolyte solution. The O^+^/R head groups define the plane of electron transfer (PET), which also includes neutral methyl spacers. The electric potential (ϕ) from the metal electrode across the film and into the bulk electrolyte is shown by the solid blue line. The potential drop between the PET and solution (ϕ_PET_–ϕ_S_) corresponds to the reduction in the driving force for electron transfer relative to a bare electrode.

Finite element simulations are based on the model in Figure 1, which schematically depicts the electric potential distribution across an interface comprising a redox-active film on a metal electrode in contact with an electrolyte solution. The redox-active site is associated with the terminal head group of the molecular film and is assumed to be irreversibly bound to the electrode surface. The model is built to approximate the structure and properties of a 2 nm thick 11-(ferrocenylcarbonyloxy)undecanethiol SAM on a metal electrode that has a theoretical full monolayer coverage of 4.5 × 10^–10^ mol/cm^2^.^18,32^ The model treats the linker molecules to the redox head group as being uncharged. The formal redox potential of the O^+^/R redox couple, *E*^0’^, is set to 0.2 V versus the potential of zero charge, *pzc*, of the bare metal (the latter, which is also equal to the solution potential, ϕ_S_, far from the electrode). Initial simulations consider the redox species in the reduced, neutral, state at a surface coverage of 1 × 10^–10^ mol/cm^2^ (sub-monolayer coverage of the redox head group is common in many experimental studies).^1,18^

The model assumes that all redox centers are located at a fixed distance, *d* = 2 nm, from the metal electrode surface (*x* = 0), identified as the plane of electron transfer (PET). Within the model, we assume the surface charge densities on both the PET and the metal electrode are delocalized. Discreteness of charge and ion-pairing is beyond the scope of this paper.^28,29,33^ The layer between the redox head groups and the metal electrode contains a dielectric region, where the hydrocarbon chains sit, and is characterized by a dielectric constant (ε_F_) equal to 7.^25^ The solvent has a dielectric constant corresponding to water (ε_S_ = 78).^34^ We assume that electrolyte ions cannot penetrate the molecular film and are thus found only in the solution beyond the PET. Γ_T_ was varied from 5 × 10^–10^, approximately full monolayer coverage,^18^ to 1 × 10^–13^ mol/cm^2^, low coverage, to mimic experiments where the redox species density is reduced by dilution with inert spacer molecules,^18^ depicted as methyl terminated species in Figure 1.

As the electrode potential, *E*(*t*), is varied during the voltammetric scan, the oxidation of R to O^+^ occurs, causing the surface coverages (mol/cm^2^) of oxidized (Γ_O_) and reduced (Γ_R_) groups to change while maintaining Γ_T_, as given by eq 1.
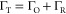
1

The fraction of the surface in the charged (oxidized state) is defined as *f* = Γ_O_/Γ_T_. Thus, the fraction of the surface in the reduced state (1–*f*) = Γ_R_/Γ_T_.

### Electrolyte Solution

In the electrolyte, the distribution of the supporting electrolyte ions and their fluxes, and the distribution of the electric potential are obtained by simultaneously solving the Nernst–Planck and Poisson equations, respectively (eqs 2 and 3, respectively), using the finite element method. The Nernst–Planck equation describes the diffusion and migration of the electrolyte ions, *i*. The Poisson equation relates the electric potential distribution to the distributions of ions in the electrolyte solution and the charges at the PET and at the electrode.
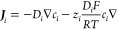
2
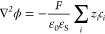
3

In eqs 2 and 3, ***J***_*i*_, *c*_*i*_, and *z*_*i*_ represent the flux, concentration, and charge number of an electrolyte ion of species *i*, respectively, while ε_0,_*T*, *F*, and *R* represent the permittivity of free space, temperature, Faraday’s constant, and the ideal gas constant, respectively. The numerical solution of eqs 2 and 3 not only provides the distributions of ions and electric potential within the electrical double layer but also the ohmic potential distribution across the bulk solution between the working and reference electrodes.

An aqueous 1:1 (perchloric acid) electrolyte is assumed,^18^ and thus, the diffusivities (*D*_*i*_) and ion mobilities of H^+^ and ClO_4_^–^ in water are used in the simulations. *D*_*i*_ for H^+^ and ClO_4_^–^ are based on literature values of 9.3 × 10^–5^ cm^2^/s and 1.8 × 10^–5^ cm^2^/s, respectively.^35^ The concentrations of the supporting electrolyte ions at the outer cell boundary of the simulation (*x* = 1 cm) were held constant at the bulk concentration of the supporting electrolyte (*c*_elec_), eq 4.
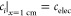
4

At the start of the voltammetric sweep, a pre-equilibration step is used to avoid any current from the initial formation of the electrical double layer. A schematic of the simulation model is presented in Supporting Information 1, Figure S1.

### Electrostatic Considerations

The interfacial electric potential (ϕ) distribution, from the electrode, across the redox film, and to the bulk electrolyte, is schematically shown by the blue curve in Figure 1. In the model, the potential far from the electrode (*x* = 1 cm) is held at the ground (ϕ_S_ = 0 V) and all electric potentials are referenced with respect to this value. This point is equivalent to the reference electrode in an experimental cell. Under zero current conditions zero current), the potential throughout the bulk solution is also equal to 0 V. However, when current is passed, such as during voltammetry, an ohmic potential drop occurs due to the finite conductivity of the electrolyte, which results in nonzero values of the potential in the solution phase. This drop is exacerbated as the electrolyte conductivity decreases. In all cases, the potential applied between the electrode, ϕ_M_, and at the reference point, ϕ_S_ (= 0 V at *x* = 1 cm) during a voltammetric experiment is defined as *E*(*t*).

Consistent with expectations of a well-ordered SAM, the electrolyte ions are not allowed to penetrate the molecular film. Thus, within the molecular film, that is, 0 < *x* ≤ *d*, the electric potential varies linearly with position and is described by the Laplace equation (eq 5).
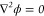
5

The charge density at the PET (*σ*_PET_) varies with the fractional coverages of the O^+^ and R redox species, eq 6.
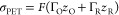
6

For the case described, the oxidized species (*z*_O_ = +1) will lead to a positive contribution to *σ*_PET_, while the neutral reduced species (*z*_R_ = 0) do not contribute. The electric fields on the film (*E⃗*_F_) and electrolyte solution (*E⃗*_S_) sides of the PET, alongside the charge density at the PET, are related by Gauss’ law, eq 7. Equation 7 emphasizes how changes in *σ*_PET_, defined by the surface coverage of redox species, eq 6, induce corresponding changes in the interfacial potential distribution.
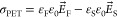
7

### Electron-Transfer Kinetics

We consider a one-electron-transfer process for the O^+^/R couple shown in eq 8
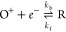
8where *k*_f_ and *k*_b_ are the potential dependent first-order electron-transfer rate constants (s^–1^) for the oxidation and reduction reaction, respectively, at the film interface. In this model, the Butler-Volmer formalization is used to describe *k*_f_ and *k*_b_, eqs 9 and 10.
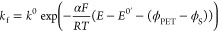
9
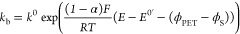
10where α is the transfer coefficient (assumed to be 0.5) and *k*^0^ is the standard rate constant (s^–1^). Importantly, eqs 9 and 10 highlight that, relative to a bare electrode, with the redox species freely diffusing, the driving force for electron transfer at the redox-active film is reduced by an amount equal to the potential drop between the PET and bulk solution, (ϕ_PET_–ϕ_S_). If both the metal and PET are treated as uncharged, as in the Nernstian model, ϕ_PET_–ϕ_S_ = 0 V and all the potential is dropped across the monolayer.

The rate of electron transfer for the O^+^/R redox couple is defined as the rate of change of the surface coverage of O^+^ with time, eq 11
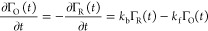
11where the rate of change of the surface coverage of R with time is equal but opposite to that of O^+^. Activities of the redox species are approximated by their respective surface coverages.^25^ Unless otherwise stated, *k*^0^ is assumed to be 1000 s^–1^, consistent with kinetic values for the SAM system shown in Figure 1.^18,32^ The effect of varying *k*^0^ is discussed in the Results and Discussion.

### Finite Element Simulations

The coupled time-dependent eqs 2, 3 and 11 were numerically solved using COMSOL Multiphysics (Version 5.6) to compute the voltammetric response. A detailed description of the mesh, boundary conditions, and numerical parameters used to solve the finite element model are included in Supporting Information S1. This model applies to 1D planar electrodes of any size. The voltammetric curves are reported as current densities. For those wishing to extend the model to microelectrodes or other electrode geometries, the COMSOL-generated model report is provided as Supporting Information, and may be followed and adapted.

## Results and Discussion

### Electrochemically Inactive Molecular Film

Before considering the case of a redox-active film, it is first useful to consider the response where the film contains no redox-active groups. Physically, this corresponds to a functionalized electrode terminated in electrochemically inactive head groups, for example, methyl groups. In this section, all parameters are as listed in the Model and Theory section, but with Γ_T_ = 0 mol/cm^2^ and *d* = 0.1, 0.2, 0.5, 1, or 2 nm. Under these conditions, the current measured in the voltammetric response is solely the result of nonfaradaic charging of the double layer (*i*_C_) at the redox inactive film–electrolyte interface.

*i*_*C*_ is proportional to the total interfacial capacitance density (*C*_T_, F/m^2^), as described by eq 12. *C*_T_ is a measure of the ability of the electrode to store charge in response to a perturbation in *E*, as described by eq 13.
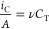
12
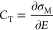
13

In eqs 12 and 13, *A* is the electrode area, ν is the scan rate and σ_M_ is the surface charge density of the electrode (C/m^2^). σ_M_ is proportional to the potential gradient across the monolayer film according to Gauss’ law,^17^eq 14.
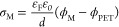
14

**Figure 2:**
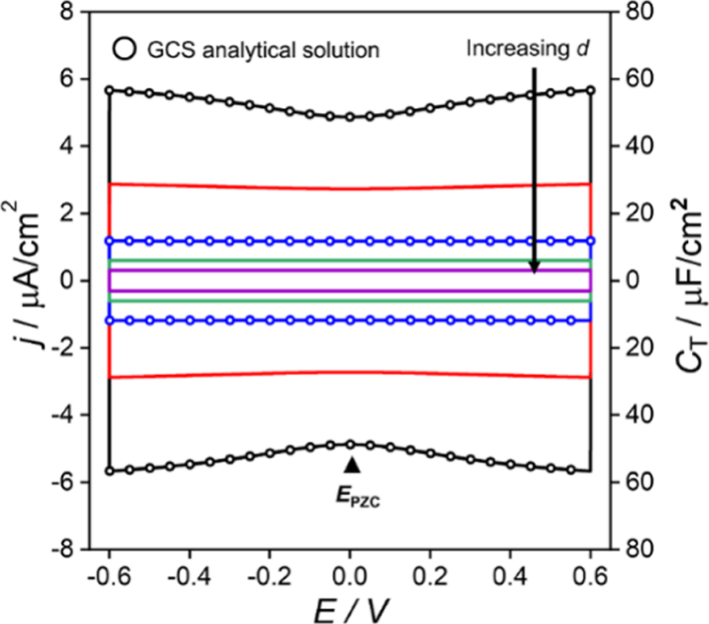
Simulated voltammetric response of an electrochemically inactive film of varying thickness, 0.1 nm (black), 0.2 nm (red), 0.5 nm (blue), 1 nm (green), and 2 nm (purple). Capacitance currents derived from the analytical solutions to Gouy–Chapman Stern theory for a Stern layer with thicknesses of 0.1 nm and 0.5 nm are shown by the circles. Simulation parameters: ε_F_ = 7, ε_S_ = 78, [HClO_4_] = 1 M, Γ_T_ = 0 mol/cm^2^, ν = 0.1 V/s, and *T* = 298.15 K.

Due to the planar geometry and absence of ions in the film, the potential gradient within the film is independent of position. Figure 2 shows the simulated nonfaradaic voltammetric response for redox-inactive films with *d* ranging from 0.1 nm (black line) to 2 nm (purple line). As *d* increases, both *C*_T_ and the nonfaradaic current density, *j*_C_ (= *i*_C_/A), decrease.

As the continuum Poisson-Nernst-Planck expressions (eqs 2 and 3) and finite element simulations treat the electrolyte ions as point charges, at large σ_M_ (corresponding to large *E* and/or small *d*), the simulations yield an unrealistically high concentration of supporting electrolyte ions at the molecular film/electrolyte interface. For example, ion concentrations of up to 30 M at this interface were obtained when *d* = 0.1 nm, as shown in Supporting Information S2, Figure S4a. More feasible interfacial concentrations (≤3 M) and capacitance values are obtained for a film thickness = 0.5 nm, as shown in Supporting Information S2, Figure S4b.^36^

The simulated data in Figure 2 were compared against the Gouy-Chapman-Stern (GCS) model (see Supporting Information S2 for calculation details), which describes the double layer structure of ions at an electrode/electrolyte interface under equilibrium conditions. It assumes that counter ions of the electrolyte can approach the electrode to a distance equal to their solvated radius, often referred to as the outer Helmholtz plane. Beyond the Helmholtz plane, electrolyte ions are thermally distributed in accordance with the Poisson–Boltzmann equation. Electrostatically, the GCS model is equivalent to the SAM model simulated here, which comprises an electrochemically inactive dielectric layer in contact with a diffuse layer. The closest approach of electrolyte ions in the diffuse layer is equal to the thickness of the film, *d*(25)*.* Analytical solutions of *C*_T_ and *j* based on the GCS model are shown by the circles in Figure 2 for the cases where *d* = 0.1 and 0.5 nm. At a moderate scan rate of 0.1 V/s, the simulated and GCS values are identical within numerical error, indicating that the simulation results at 0.1 V/s also correspond to equilibrium conditions. As shown later, one advantage of the finite element simulations, relative to the GCS model, is that they allow for the calculation of the nonequilibrium ion and potential distributions that are obtained at higher scan rates. This capability is not feasible with the GCS theory.

In all the *C*_T_ vs *E* curves shown in Figure 2, a minimum in the interfacial capacitance density at *E* = 0 V is present, also consistent with the GCS model. However, the minimum is only clearly visible for the cases where *d* ≲ 0.2 nm. For the electrochemically inactive, uncharged film, this minimum occurs at the *E*_pzc_. At this potential, there is no charge stored on the electrode surface and the electric field within the film is 0. The *C*_T_ for all film thicknesses is shown in Figure 2 following the expected linear proportionality to 1/*d*, when sufficiently far from the *E*_pzc_. The *C*_T_ value calculated for the 2 nm film (∼3 μF/cm^2^) is slightly larger than the values reported in the literature (1–2 μF/cm^2^).^1,37^ This is due to our choice of ε_F_ (=7) being slightly larger than those reported for electrochemically inactive films, where ε_F_ has been estimated as ∼ 2.6.^37^

### Reversible Electron Transfer—O^+^/R Film

In this section, we consider the simulated voltammetric response under reversible electron transfer conditions, employing the full electrostatic model described above. These conditions typically correspond to moderate scan rates (<1 V/s) and solutions containing a high concentration of supporting electrolyte in the bulk (>0.1 M), resulting in negligible ohmic potential drop. Initially, *E* is set to negative of *E*^0’^, corresponding to the fully reduced state, *f* = 0.

For a reversible reaction, the driving force (see eqs 9 and 10) for electron transfer at the PET is related to the surface coverages of O^+^ and R, eq 15. All parameters are previously defined.
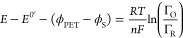
15

The inclusion of a redox-active head group in the model makes it necessary to consider the current contribution due to electron transfer between the metal electrode and the redox center located at the PET. For the O^+^/R redox couple, when *E* is scanned at a constant scan rate, the faradaic current (*i*_F_) is defined by the rate of change of the surface coverage of the O^+^ group, as stated by eq 16
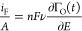
16

The total current (*i*_T_) passed represents the sum of faradaic and nonfaradic (capacitive) charging current contributions, eq 17.
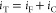
17

**Figure 3:**
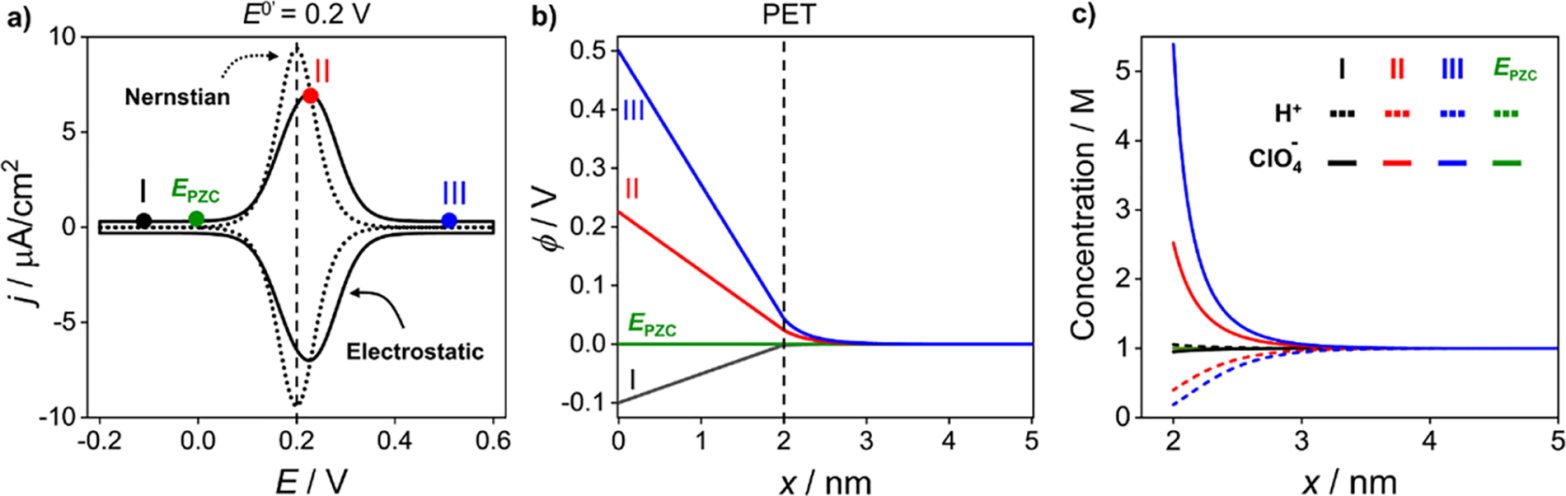
**(**a) Simulated voltammetric responses of a redox-active (O^+^/R) film produced using the Nernstian model (dotted) and electrostatic model (solid) at a scan rate of 0.1 V/s. (b) Plot of the interfacial potential distribution and (c) electrolyte concentration (H^+^ and ClO_4_^–^) vs distance from the electrode surface (*x*) at characteristic potentials throughout the voltammetry, as labeled on part a. Simulation parameters are *d* = 2 nm, ε_F_ = 7, ε_S_ = 78, [HClO_4_] = 1 M, Γ_T_ = 1 × 10^–10^ mol/cm^2^, *E*^0’^ = 0.200 V vs **ϕ**_S_ (= 0 V), *k*^0^ = 1000 s^–1^, *ν* = 0.1 V/s, *T* = 298.15 K, I (*E* = −0.100 V), II (*E* = 0.226 V), and III (*E* = 0.500 V).

Figure 3a shows the voltammetric response of the redox-active SAM with *E*^0’^ = 0.2 V under two conditions. The first, (i) (ϕ_PET_–ϕ_S_) = 0 V, corresponds to the absence of electric double layer effects on the faradaic response (Nernstian response). Under these conditions, the *i–E* response can also be predicted analytically.^17^ The resulting simulated curve (dotted line) in Figure 3a has an fwhm of 90.6 mV at (25 °C) with the peak current occurring at *E*_p_ = *E*^0’^ = 0.2 V. Both values are in agreement with those obtained analytically.^17^ The second, (ii) (ϕ_PET_–ϕ_S_) has a finite value arising from the electric charge on the electrode and O^+^ head groups. When the charge of the redox head groups and charge on the metal electrode is considered (eqs 6 and 14 respectively), electrostatic interactions between the charged O^+^ species and electrolyte ions must also be accounted for. This results in not all of the potential being dropped across the redox film, due to the nonzero (ϕ_PET_–ϕ_S_) value. The solid black line in Figure 3a shows the simulated voltammetric response under these conditions. As can be seen, whilst the cathodic and anodic peaks are still mirrored images of each other, the fwhm has broadened to 133 mV and both *E*_p_ values are shifted positive of *E*^0’^ by ∼25 mV.

To understand the physical origin of the shape and shift in *E*_p_ of the voltammetric response when electrostatic interactions are present, it is useful to consider how the distribution of ϕ (Figure 3b) and the supporting electrolyte ions (Figure 3c; ClO_4_^–^ solid, H^+^ dashed) change with *E*. These profiles are plotted as a function of distance, *x*, from the electrode surface, for four different potentials during the voltammetric scan: (I) *E* = −0.100 V at which the redox film is in the fully reduced and uncharged state (*f* = 0); (II) *E* = 0.226 V, a potential corresponding to *E*_p_; (III) *E* = 0.500 V, a potential at which the film is fully oxidized and in the positively charged state (*f* = 1); and (IV) *E* = *E*_pzc_ = 0.000 V.

For the profiles shown in Figure 3b, the applied potential decays linearly, from the specified *E*(ϕ) value across the molecular film. For I, the film is in the reduced state and thus represents an uncharged film (σ_PET_ = 0); ϕ_PET_–ϕ_S_ = 0. Under these conditions, the potential at the molecular film/electrolyte interface is determined only by the surface charge density on the electrode, which here is negative. As *E* is swept positively from I to II and II to III, the film charge state increases as more O^+^ groups are created, which in turn increases both *σ*_PET_ and *ϕ*_PET_. The increased positive surface charge density at the PET results in an accompanying decrease in the electric potential drop across the film, by an amount (ϕ_PET_–ϕ_S_), compared to the uncharged state. This results in the terminal redox head groups seeing a reduced driving force compared to the situation where electrostatic interactions are absent. In turn, a greater electrode polarization is required to oxidize the film, hence the positive shift in *E*_p_ and peak broadening. Decreasing either *c*_elec_ or the thickness of the film results in a broader and more positively shifted voltammogram, due to an increase in ϕ_PET_–ϕ_S_, the proportion of the electric potential which is dropped within the electrolyte solution. For IV, at *E* = *E*_pzc_ = 0 V, ϕ_PET_ = 0 V and no electric field exists within the film (0 < *x* ≤ 2 nm).

In the diffuse layer, the remaining electric potential decays approximately exponentially from ϕ_PET_ to ϕ_S_, over a distance of ∼3 nm from the PET. The electric potential decay is different for each applied *E* value and reflects differences in the distribution of H^+^ and ClO_4_^–^ in the diffuse layer, as shown in Figure 3c. In the diffuse layer, the electrolyte ions redistribute in order to screen the excess charge on the metal and at the PET and maintain electroneutrality. For example, when the sum of the surface charge density of the metal electrode and PET is positive, the diffuse layer will counterbalance this positive charge by an accumulation of ClO_4_^–^ (solid lines) and depletion of H^+^ (dashed lines) relative to their concentration in bulk solution, Figure 3c. This can be seen for electrode potentials II (red) and III (blue), where the film is either nearly half or fully oxidized. Both cases correspond to a positive charge density on the metal and at the PET, increasing the concentration of ClO_4_^–^ in the diffuse layer. For electrode potential I, where the redox film is in the uncharged state and thus there are no charged species at the interface, the negative surface charge density on the electrode leads to a small accumulation of H^+^ in this region, Figure 3c.

**Figure 4:**
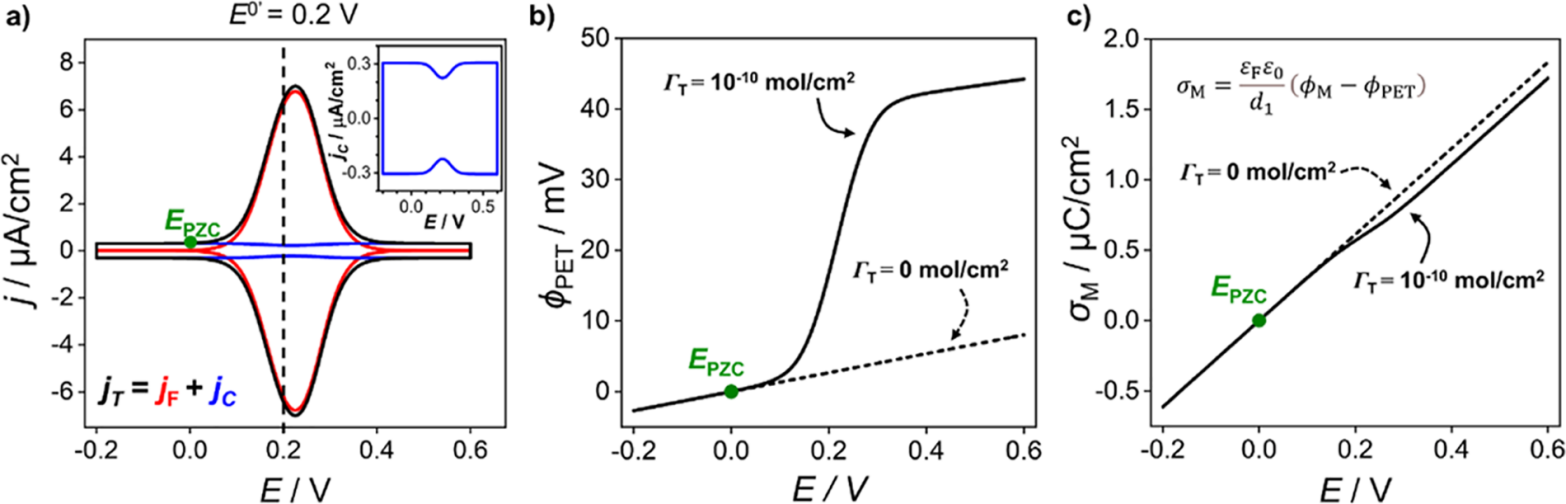
**(**a) Faradaic (red) and nonfaradaic (blue) contributions to the simulated total current density (black) during voltammetry of a 2-nm thick redox-active (O^+^/R) film at 0.1 V/s (inset: zoom in of the capacitive contribution). (b) Electric potential at the PET when Γ_T_ = 10^–10^ mol/cm^2^ (solid) and Γ_T_ = 0 mol/cm^2^ (dashed) and the corresponding (c) surface charge density on the metal electrode for Γ_T_ = 10^–10^ mol/cm^2^ (solid) and Γ_T_ = 0 mol/cm^2^ (dashed). Simulation parameters as in Figure 3 unless otherwise stated.

The area under the faradaic peak for a redox-active monolayer is often used to determine the surface coverage of redox groups.^16^ For these measurements, the baseline charging current is nearly always assumed constant, that is, potential independent, in the faradaic region of interest. Figure 4a shows the nonfaradaic (capacitive) component of the voltammetric response for the redox-active film (blue line). Also shown are the responses for the faradaic current only (red line) and the total current (black line). The nonfaradaic current density displays a small dip in current (shown in more detail in the inset to Figure 4a) that is close to the peak potential of the faradaic response. This dip leads to a very small underestimation of the charge associated with the true surface coverage, when a constant nonfaradaic current is assumed. In the case shown in Figure 4a, the background-subtracted faradaic peak current (assuming constant background) is ∼1% less than the true background-subtracted faradaic response. The situation is exacerbated by reducing *c*_elec_ and/or using thinner redox films, lower surface coverages of the redox head group, and films with larger ε_F_ values. An example of a worst-case scenario is provided in SI 3, for a film with Γ_T_ = 1 × 10^–12^ mol/cm^2^ and *d* = 0.75 nm; here, the error in Γ_T_ increases to ∼17%, when a constant background is assumed.

The origin of the dip in the capacitive current density can be understood by considering the data shown in Figure 4b,c, alongside eq 13. The charge stored on the electrode is related to the electric potential drop across the redox film by eq 14 and is therefore dependent on ϕ_PET_, which is plotted versus *E* in Figure 4b. The nonfaradaic current density plotted in the inset of Figure 4a is derived from the dependence of σ_M_ in response to a change in *E* and is equal to the gradient of the σ_M_ vs. *E* lines shown in Figure 4c, eq 13*.* For the electrochemically inactive monolayer case where Γ_T_ = 0 mol/cm^2^, the interfacial potential at the PET changes linearly by a total of 10 mV across the potential range −0.2 to+0.6 V. For a redox-active monolayer (Γ_T_ = 10^–10^ mol/cm^2^) the change in ϕ_PET_ with *E* matches that of the electrochemically inactive monolayer ^2^ when the redox film is in the uncharged, reduced state. However, as the potential increases further and induces oxidation of the film, the ϕ_PET_ rises sharply near *E*^0’^ and then increases at a similar rate to that seen in the reduced state. Across the potential range encompassing full oxidation of the film, ϕ_PET_ varies by 48 mV, with the greatest charge seen at *E*_p_. Overall, this leads to a decrease in the potential gradient within the film, and thus a reduction in σ_M_ near *E*_p_, as shown in Figure 4c. These results demonstrate the interdependence of the faradaic processes and electric potential distribution.

### Redox Group Surface Coverage

**Figure 5:**
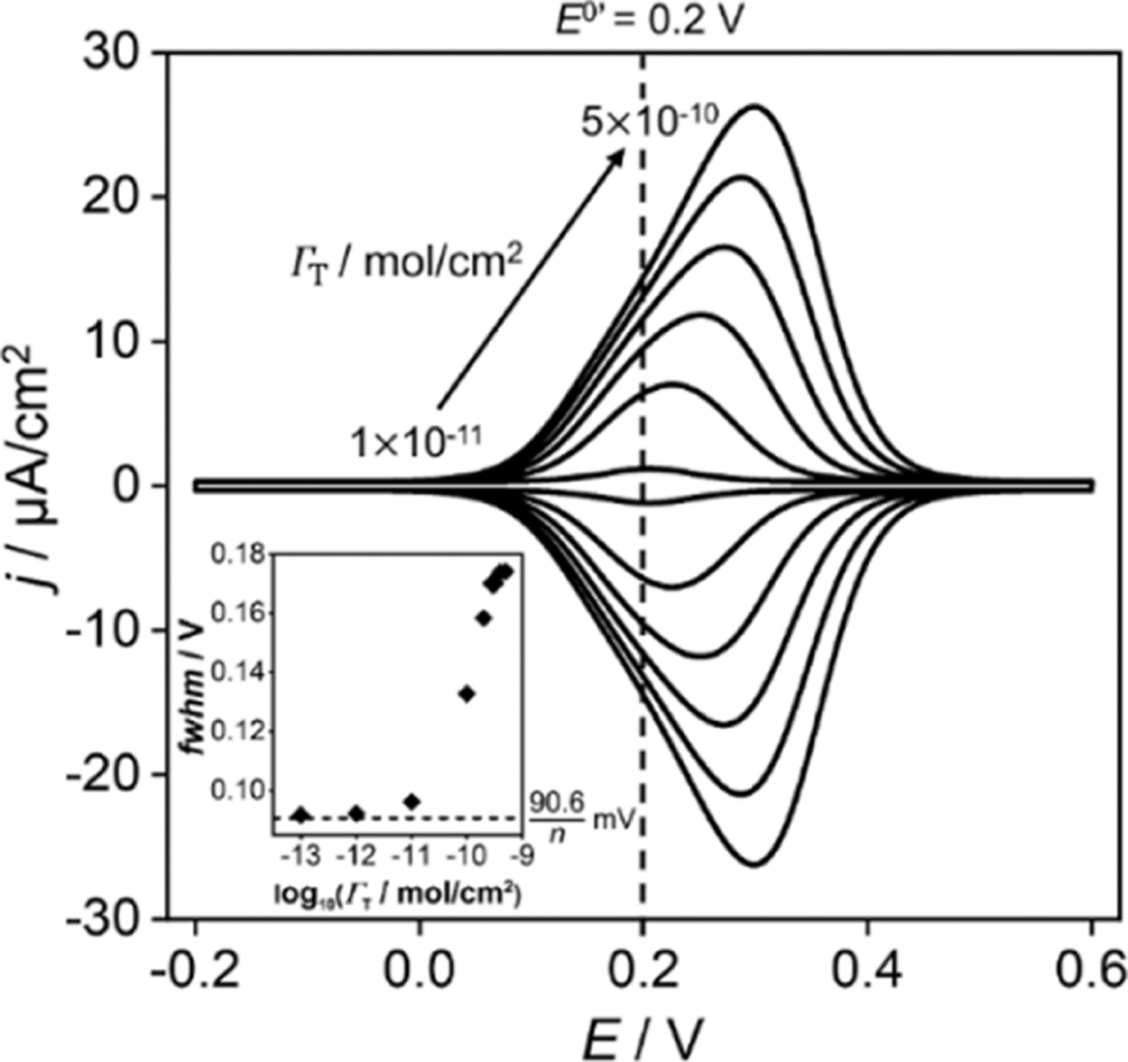
Simulated voltammetric responses of a redox-active (O^+^/R) self-assembled monolayer when the surface coverage (Γ_T_) of redox groups is varied between 1 × 10^–11^ and 5 × 10^–10^ mol/cm^2^ at 0.1 V/s (coverages shown 10^–11^ and 10^–10^, 2 × 10^–10^, 3 × 10^–10^, 4 × 10^–10^, and 5 × 10^–10^ mol/cm^2^). The calculated fwhm vs Γ_T_ is provided in the inset for these voltammograms and those at coverages of 10^–13^ and 10^–12^ mol/cm^2^. Except for the surface coverage of the redox head groups, all other parameters are as listed in the caption of Figure 3.

Experimentally, the surface coverage of redox head groups can be varied from full monolayer to zero by dilution with alkylthiol molecules terminated in methyl groups, as shown in Figure 1.^1^ Increasing the surface coverage results in larger surface charge densities at the PET, eq 6. Simulated voltammograms for monolayer surface coverages between Γ_T_ = 5 × 10^–10^ and Γ_T_ = 1 × 10^–11^ mol/cm^2^ are shown in Figure 5. Simulations for lower surface coverages of 1 × 10^–12^ and 1 × 10^–13^ mol/cm^2^ are provided in SI 4. The inset shows how the peak fwhm varies with Γ_T._

As the voltammograms and inset show, increasing Γ_T_ leads to a broader fwhm, a more asymmetric voltammogram, and a positive shift away from *E*^0’^. The outputs of our model are in qualitative agreement with the experimental results from redox SAMs.^18,38−42^ The wave asymmetry results from an increase in surface charge in the headgroups, and thus a more positive electric potential at the PET, as the film is converted to O^+^. This effect is equivalent to electrostatic repulsion between the O^+^ head groups and results in an increasing overpotential. However, the anodic and cathodic branches of the voltammograms remain mirrored images of each other, reflecting equilibrium conditions for both the electron-transfer reaction and establishment of the electric double layer, at this moderate scan rate (0.1 V/s). Three different regions of behavior are observed. At low surface coverages (<1 × 10^–12^ mol/cm^2^) (as shown in SI 4) the peak is centered at *E* = *E*^0’^ and the fwhm approaches a constant value of 92 mV but does not quite reach the theoretical value of 90.6 mV for a one-electron transfer Nernstian process. In the intermediate region, Figure 5, between 1 × 10^–11^ mol/cm^2^ and 1 × 10^–10^ mol/cm^2^, the fwhm *increases* from 96 mV to 133 mV and *E*_p_ shifts positively by ∼25 mV. Further increases in Γ_T_ result in a continued positive shifting of *E*_p_ but with a less dramatic increase in the fwhm and wave asymmetry. At the highest surface coverage of 5 × 10^–10^ mol/cm^2^, fwhm = 174 mV, with a peak shift of ∼100 mV.

The presence of the O^+^/R couple at the PET introduces a positive *σ*_PET_ when the film is in the O^+^ state. The accompanying increase in *ϕ*_PET_ means that the electric potential drop in the film (ϕ_M_–ϕ_PET_) is reduced making electron transfer less thermodynamically favorable. At lower Γ_T_, the magnitude of *σ*_PET_ generated when the film is in the O^+^ state is very small, leading to a small reduction in (ϕ_M_–ϕ_PET_), resulting in an fwhm that is closer to the Nernstian value of 90.6/*n* mV.

### Finite Electron Transfer Kinetics

In practical situations, *k*^0^ will vary based on factors such as the film thickness and chemical functionality of the linker chain used to tether the redox head group to the metal electrode.^16^ We now consider the effect of electron-transfer kinetics by simulating voltammograms in which *k*^0^ is systematically varied from 0.01 to 10,000 s^–1^. The scan rate of 0.1 V/s and [HClO_4_] of 1 M were maintained alongside all other parameters discussed in the previous sections unless otherwise stated.

**Figure 6:**
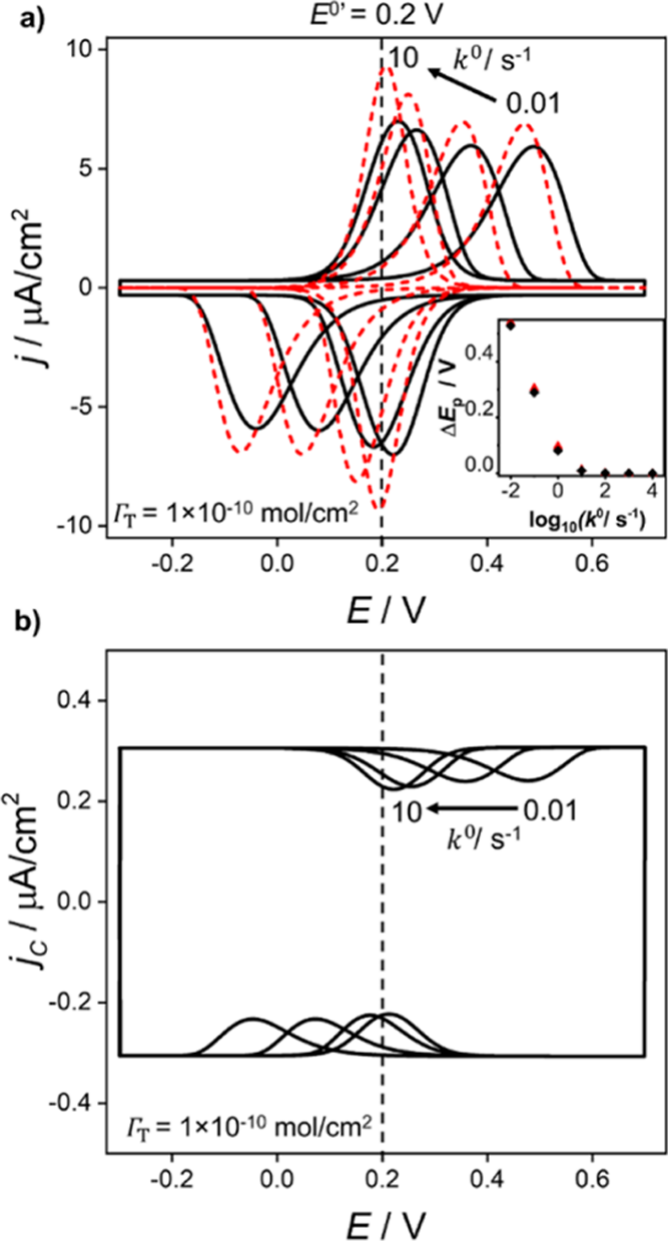
Simulated (a) total and (b) nonfaradaic voltammetric responses of a redox-active (O^+^/R) film for *k*^0^ = 0.01, 0.10, 1.0, and 10 s^–1^. Red dashed curves correspond to the voltammetric response in the absence of electrostatic effects, where the waveshape reflects only the influence of electron-transfer irreversibility. Black curves include the effect of electrostatics and electron-transfer irreversibility. Voltammetric responses for *k*^0^ = 100, 1000, and 10,000 s^–1^ are not shown, as these display negligible peaking splitting, Δ*E*_p_. Inset in (a): plot of Δ*E*_p_ as a function of log(*k*^0^). Except for *k*^0^, model parameters are as listed in the caption of Figure 3.

Figure 6a shows simulated voltammograms for *k*^0^ between 0.01 and 10 s^–1^. The red dashed curves are computed by neglecting electrostatic contributions at the molecular film–electrolyte interface, while the black curves include the full electrostatic description of the double layer, as presented in the Model and Theory section. Thus, a comparison of the red and black curves at constant *k*^0^ allows visualization of the influence of the interfacial potential distribution. The peak separation (Δ*E*_p_) between the anodic and cathodic peak potentials is shown in the inset of Figure 6a. For *k*^0^ > 10 s^–1^, the cathodic and anodic waves are symmetric and mirror images of each other, with Δ*E*_p_ ∼ 0, indicating that reversibility is maintained. For *k*^0^ < 0.1 s^–1^, the voltammetric waves become asymmetric with Δ*E*_p_ increasing ∼100 mV per decade decrease in *k*^0^.

The peak splitting shown in Figure 6a is predominately due to finite electron-transfer kinetics. However, the electric double layer still plays a role. Specifically, Δ*E*_p_ is slightly smaller when compared to the voltammograms that do not include electrostatic interactions (red curves). For example, at the lowest *k*^0^ value of 0.01 s^–1^, Δ*E*_p_ is 528 mV when electrostatics are included compared to 542 mV without electrostatics, a difference of 14 mV. As can be seen from Figure 6b, the dip in the nonfaradaic current density discussed in Figure 4, follows the faradaic response, shifting away from *E*^0’^ toward the location of the individual cathodic and anodic peak currents. The dips in the cathodic and anodic capacitive currents also become asymmetric due to the interdependence of the capacitance on the charge state of the redox head groups.

Whilst Ohtani also previously modeled the effect of finite electron-transfer kinetics on the voltammetric response of a redox-active film,^29^ they assumed an equilibrium structure for the diffuse double layer. At the low scan rates employed in Figure 6 (in conjunction with the high supporting electrolyte conditions) it is reasonable to assume both their and our model will produce the same result. However, the Ohtani model cannot capture the physical processes taking place when the net flux of ions is no longer negligible, as is the case for much higher scan rates and/or low concentrations of electrolyte. Therefore, Ohtani does not describe nonequilibrium physical processes such as ohmic potential drop.

### Ohmic Drop and Mass Transport

The ohmic potential drop in the bulk solution, *iR*_u_, where *R*_u_ is the uncompensated solution resistance, can be significant under conditions of high scan rates (*ν*) and/or high surface coverages (higher currents) and/or decreased *c*_elec_ (larger *R*_u_). Here, we consider how varying the scan rate from 0.01 V/s to 1000 V/s and *c*_elec_ from 0.01 M to 1 M impacts the structure of the double layer, ohmic drop, and wave shape. In these simulations, *k*^0^ is set to 10^7^ s^–1^ to prevent complications arising from slow electron-transfer kinetics. Thus, all nonidealities observed in the voltammetric waveshape reflect solely the effects of the electric potential and ion distribution across the monolayer and bulk solution.

As in a real electrochemical cell, the *iR*_u_ drop depends upon the distance between the working electrode and reference electrodes.^43^ In our simulations, we chose this distance to be 1 cm, which is a reasonable assumption in real experiments. For the purpose of simulating the effects of a finite *R*_u_, we note that the product of the current density, *j*, and *R*_u_, that is, *jR*_u_, is independent of the electrode size. This results from the assumption that the working and reference electrodes are both planar, of equal area, parallel to each other, and that the ionic current path between the two electrodes is always orthogonal to both electrodes. Thus, specific values of *R*_u_ are not specified. For exactly analogous reasons, the charging time constant, *R*_u_*C*_T_, is also independent of the electrode size. Details of calculating *iR*_u_ and *R*_u_*C*_T_ based on the electrolyte ion mobilities and simulation geometry are presented in SI 5 and SI 6, respectively.

**Figure 7:**
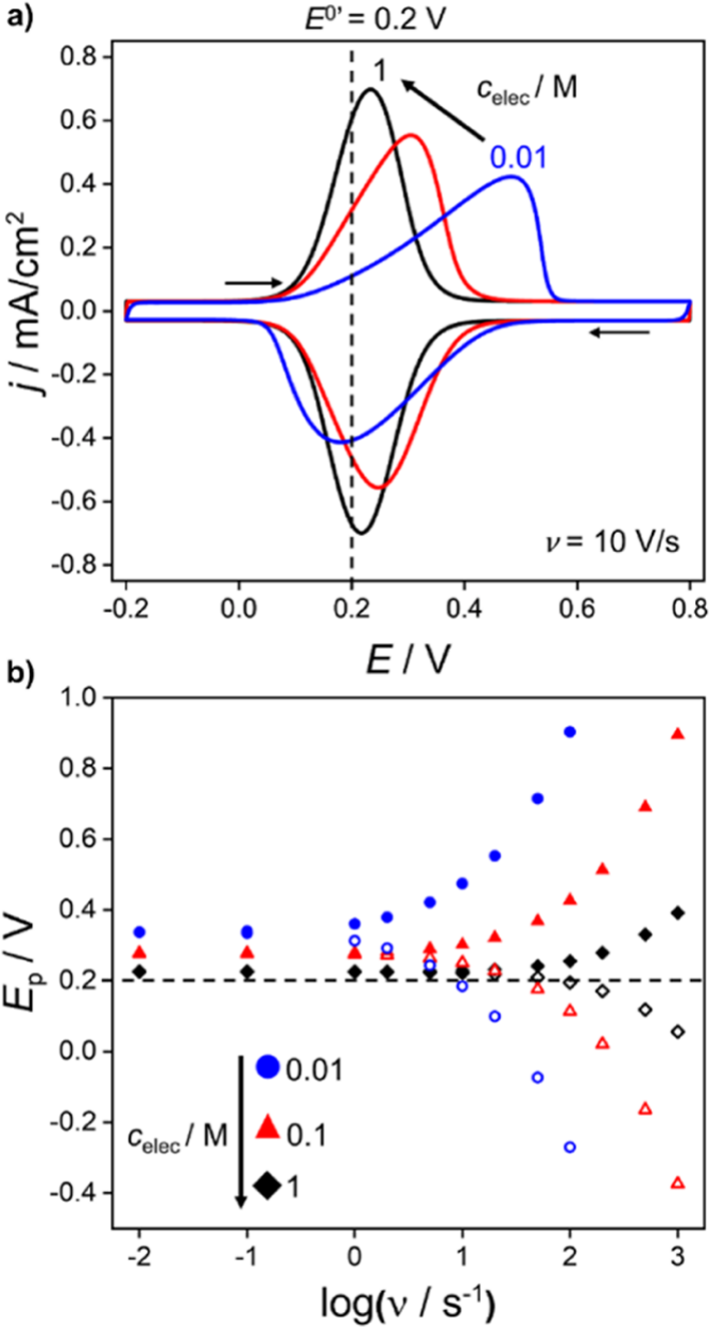
(a) Voltammetric response at 10 V/s for a redox-active (O^+^/R) film corresponding to *c*_elec_ = 1 M (black), 0.1 M (red), and 0.01 M (blue). (b) Plot of the anodic and cathodic peak positions for *ν* ranging from 0.01 to 1000 V/s. All data correspond to *k*^0^ = 10^7^ s^–1^. Other parameters as in Figure 3. No peak splitting is observed in simulations in the absence of consideration of the electric double layer and *iR*_u_ drop in bulk solution [dashed line in part (b)].

The influence of *c*_elec_ on the shape of the voltammetric response is seen in Figure 7a for a high scan rate of 10 V/s. In general, as *c*_elec_ is decreased, the cathodic and anodic peak splitting increase, the peaks broaden, and the wave shifts positively. This behavior is most evident at 0.01 M, where the reduced supporting electrolyte concentration leads to a larger proportion of *E* being dropped across the solution phase, decreasing the driving force for electron transfer. The structure of the double layer in combination with a finite solution resistance at 10 V/s gives rise to markedly different current–voltage responses. In Figure 7b the scan rate dependence of the anodic and cathodic peaks (*E*_p_) is plotted as a function of *c*_elec_. The full range of voltammograms, from which the data shown in Figure 7b are derived, are displayed in Supporting Information 7, Figure S9. In Figure 7b, the dashed line corresponds to the Nernstian response, with *E*^0’^ = 0.2 V. At low scan rates (<0.1 V/s) the anodic and cathodic peaks occur at the same potential, that is, there is no peak splitting. However, as the scan rate is increased, the increase in peak splitting is the result of ohmic potential loss across the electrolyte solution (*vide infra*).

**Figure 8:**
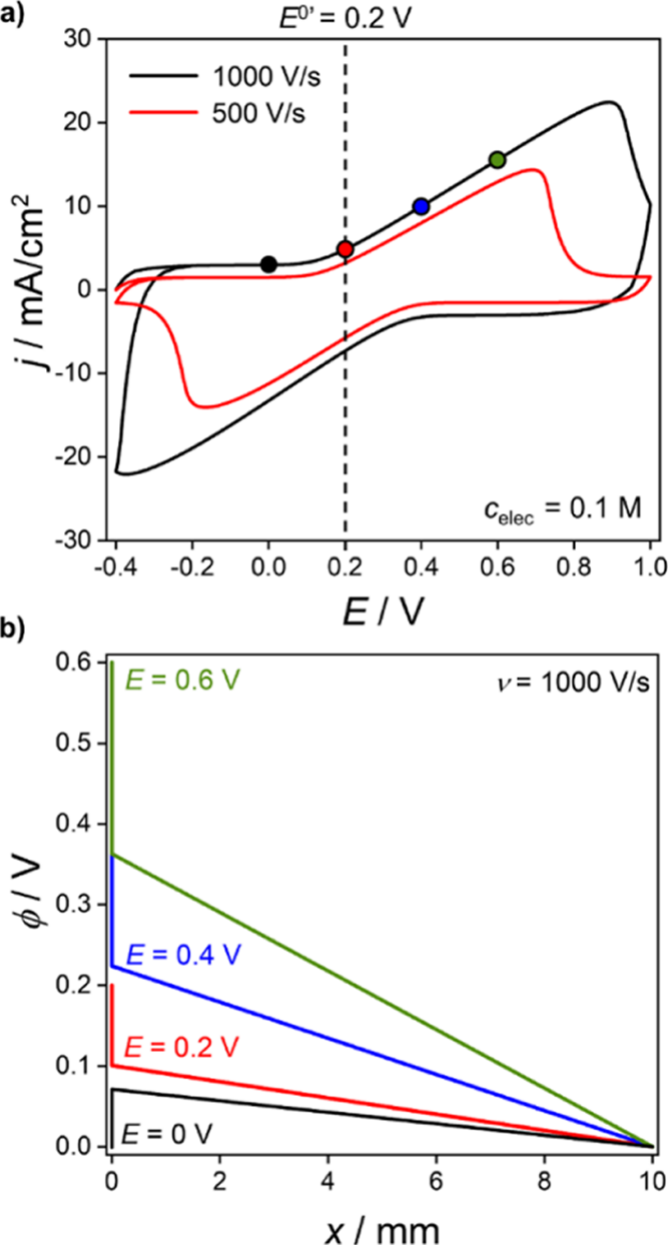
(a) Simulated voltammograms for a redox-active (O^+^/R) film with *c*_elec_ = 0.1 M, at ν = 500 (red) and 1000 V/s (black). (b) Electric potential distribution between the working (*x* = 0) and reference (*x* = 1 cm) electrodes. All data correspond to *k*^0^ = 10^7^ s^–1^. Other parameters as in Figure 3.

Simulated voltammograms at 500 and 1000 V/s in 0.1 M electrolyte solution are presented in Figure 8a. At these scan rates, the anodic and cathodic peaks become very distorted by the *iR*_u_ drop in solution. Figure 8b displays the electric potential profile as a function of the distance from the working electrode to the reference electrode, at 1000 V/s in 0.1 M electrolyte. Figure 8b shows that a large fraction of *E* is dropped in the region between just outside the electric double layer and the reference electrode. Electric potential versus distance plots for a wider range of *c*_elec_ and scan rates are shown in Supporting Information 7, Figure S10, at *E* = *E*^0’^ = 0.2 V (forward scan). Also given in Figure S10 are the corresponding plots of simulated electrolyte concentration versus distance. The linear electric potential profiles in the bulk solution region, shown in Figure 8b and S10, clearly indicate that this potential loss is due to the solution resistance. At the high scan rates employed in Figure 8, a transient *R*_u_*C*_T_ charging component (SI 6) is also visible at the end of range switching potentials (and in Figure S9b,c). In Figure 7a, simulated at 10 V/s, the charging occurs too quickly to be seen at *c*_elec_ of 1 and 0.1 M. A lower *c*_elec_ (higher *R*_u_) of 0.01 M is required to increase the time constant sufficiently such that a charging response is now visible.

Both Laviron and Nicholson derived methods for obtaining *k*^0^ from the position of the cathodic and anodic peaks for a particular scan rate.^44,45^ Both methods assume that the voltammetric response is purely due to finite electron-transfer kinetics and have been frequently applied to redox film voltammetry to extract *k*^0^(2,14)^.^ However, our simulations show that even at 1 M supporting electrolyte concentration and *k*^0^ = 10^7^ s^–1^, for *ν* > 10 V/s^–1^, a non-negligible shift in peak splitting results solely from the ohmic potential drop. Peak splitting resulting from ohmic drop appears very similar to that resulting from slow electron-transfer kinetics. The former could easily be mistaken for the latter, introducing errors into measurements of *k*^0^. Notably, as shown, the peak splitting in Figures 7 and 8 (and Figures S9 and S10) are exacerbated at lower *c*_elec_ and higher *ν*.

Finally, so far, we have only considered the O^+/^R redox couple. However, the finite element simulations can readily be extended to other redox systems. SI 8 details how the voltammetric and interfacial potential profiles change when considering O^–^/R^2-^ (*n* = 1) and O^+^/R^–^ (*n* = 2) redox films.

## Conclusions

In this work, we have developed a finite element model that simulates the electric potential distribution across the entire cell during voltammetry of monolayer redox film electron-transfer redox reactions. Our model, which provides a means to compute the driving force for electron transfer at the PET, explicitly accounts for the coupling of ion transport in the bulk solution with the dynamic redistribution of ions within the diffuse layer during the voltammetric scan. In this way, electrostatic effects at the molecular film/electrolyte interface, which are present due to the changing charge states of the redox head groups during voltammetry can be appropriately accounted for. This new development also allows the simulation of electrochemical behavior under conditions where ohmic potential losses are significant, and the electric double layer is no longer described, even qualitatively, by the GCS (or any equilibrium) model of the electric double layer. The model has been generalized to include the effect of slow-electron transfer.

This model represents the most comprehensive treatment of a redox film electrode system to date, and can be readily applied to a wide range of redox film electron-transfer reactions, including systems with multiple redox-active surface species, in order to determine *k*^0^ accurately. Furthermore, the use of finite element modeling makes the model much more widely accessible to the experimentalist than previous analytical approaches. The interdependence of the faradaic and nonfaradaic current signals is not unique to this planar redox film electrode system, but applies equally to other situations, for example, microelectrode redox film electrodes and soluble solution species undergoing electron transfer. Using the supplied COMSOL-generated report, the interested experimentalist can either adapt the model or use it as is, depending on their system of interest. We note that the well-known limitation of treating ions as point charges, as is done in the GCS model, applies equally to our predictions from finite element modeling.

Simulated voltammograms for redox systems with very large electron-transfer rates, e.g. *k*^0^ = 10^7^ s^–1^, scanned at fast scan rates (>10 V/s), demonstrate that peak splitting arises from ohmic losses even when the solution contains a high concentration (>0.1 M) of supporting electrolyte. This distortion of the wave closely mimics the effect of slow electron transfer. Thus, the application of Laviron *ΔE*_p_ vs. log(ν) type plots for the measurement of *k*^0^ requires caution to ensure that ohmic losses do not lead to underestimation of *k*^0^ values. We advocate for use of this model instead.

## Supporting Information Available

COMSOL-generated report corresponding to the finite element model (PDF)
Details of the finite element model implemented in COMSOL Multiphysics; voltammetric response of electrochemically inactive molecular films; quantification of experimental errors caused by incorrect assumptions on the behavior of the nonfaradaic current; simulated voltammetry of electrochemically active molecular films with low surface coverages of redox-active groups; calculating the uncompensated resistance of the electrochemical cell; *R*_u_*C*_T_ transient response at the switching potential; influence of supporting electrolyte and scan rate on the voltammetric response; and O^–^/R^2-^ and O^+^/R^–^ Redox Monolayers (PDF).
