# Supplementary material for: Finite Element Modeling of the Combined Faradaic and Electrostatic Contributions to the Voltammetric Response of Monolayer Redox Films
Source: Anal Chem. 2022 Sep 7;94(37):12673–82. doi: 10.1021/acs.analchem.2c01976 (PMC9494304; doi:10.1021/acs.analchem.2c01976)
Supplement: Supplementary file 1 — ac2c01976_si_001.pdf [file ac2c01976_si_001.pdf]

Finite Element Modelling of the Combined Faradaic and Electrostatic Contributions to the Voltammetric Response of Monolayer Redox Films

Katherine J. Levey,[a,b] Martin A. Edwards,[c] Henry S. White,*[d] and Julie V. Macpherson*[a,b]

[a] Department of Chemistry and [b]Centre for Diamond Science and Technology, University of Warwick, Coventry, CV4 7AL, UK

[c] Department of Chemistry & Biochemistry, University of Arkansas, Fayetteville, AR 72701, USA

[d] Department of Chemistry, University of Utah, 315S 1400E, Salt Lake City, UT 84112, USA

Contents

1 Global Definitions 3
1.1 Parameters 3
2 Component 1 6
2.1 Definitions 6
2.2 Geometry 1 8
2.3 Transport of Diluted Species 9
2.4 Electrostatics 14
2.5 Surface Reaction on PET 23
2.6 Mesh 1 28
3 Study 1 32
3.1 Parametric Sweep 32
3.2 Time Dependent 32
3.3 Solver Configurations 33
4 Results 35
4.1 Datasets 35
4.2 Plot Groups 37

1 Global Definitions

USED PRODUCTS

COMSOL Multiphysics
Chemical Reaction Engineering Module

COMPUTER INFORMATION

CPU	Intel64 Family 6 Model 158 Stepping 9, 4 cores
Operating system	Windows 10

1.1 PARAMETERS

1.1.1 General System & Geometry

GENREAL SYSTEM & GEOMETRY

**Name**	**Expression**	**Value**	**Description**
cAnion_b	cHb	1000 mol/m³	Bulk anion concentration
cCation_b	cClO4_b	1000 mol/m³	Bulk cation concentration
cHb	1 [M]	1000 mol/m³	
cClO4_b	cHb	1000 mol/m³	
d1	2 [nm]	2E−9 m	Length of alkanethiol
L	1 [cm]	0.01 m	Length of domain
D_H	9.311*10^ - 5 [cm^2/s]	9.311E−9 m²/s	Diffusion coefficient for a proton
D_ClO4	1.792*10^ - 5 [cm^2/s]	1.792E−9 m²/s	Diffusion coefficient for ClO4-
z_Anion	-1	−1	Charge of the anion
z_Cation	+1	1	Charge of the cation
T	298.15[K]	298.15 K	
D_Anion	D_ClO4	1.792E−9 m²/s	Diffusion coefficient for ClO4-

Name	Expression	Value	Description
D_Cation	D_H	9.311E−9 m²/s	Diffusion coefficient for a proton
RT_nF	((R_const*T)/(n*F_const))	0.025693 V	
Kappa	e_const*sqrt((2*cClO4_b*N_A_const)/(epsilon_s *epsilon0_const*k_B_const*T))	3.2978E9 1/m	
DebyeLength	1/Kappa	3.0324E−10 m	Debye length

1.1.2 Redox Film

REDOX FILM

Name	Expression	Value	Description
Gamma_T	1*10^ - 10 [mol/cm^2]	1E−6 mol/m²	(Total Surface Coverage)
k0	1000 [s^ - 1]	1000 1/s	Standard rate constant (set to be large)
alpha	0.5	0.5	
epsilon_1	7	7	For the alkane chain layer
epsilon_s	78	78	Assuming 298 K for water
E0	0.2[V]	0.2 V	E0 for the 1e transfer R/O+ couple
n	zOx - zRed	1	
zOx	+1	1	Charge of the oxidized species
zRed	0	0	
nF_RT	(F_const)/(R_const*T)	38.922 1/V	

1.1.3 Mesh

MESH

Name	Expression	Value	Description
Max_PET	1E-10 [m]	1E−10 m	
Max_F	2E-10 [m]	2E−10 m	
film_points	1E-10 [m]	1E−10 m	

1.1.4 Cyclic Voltammetry

CYLIC VOLTAMMETRY

Name	Expression	Value	Description
OpV_sw	0.6 [V]	0.6 V	Switching overpotential
OpV_i	-0.2 [V]	−0.2 V	Initial overpotential

Name	Expression	Value	Description
SegmentDuration	ScanRange/v	8 s	
ScanRange	abs(OpV_i - OpV_sw)	0.8 V	
v	0.1 [V/s]	0.1 V/s	Scan rate
sampling	1 [mV]	0.001 V	Potential step size of the CV
sampling_time	2.5*SegmentDuration/(((abs(OpV_i - OpV_sw)*2.5))/(sampling))	0.01 s	Corresponding sampling time interval for the potential step size at the specified scan rate

2 Component 1

SETTINGS

Description	Value
Unit system	Same as global system (SI)

2.1 DEFINITIONS

2.1.1 Variables

Surface Reaction - 1e

SELECTION

Geometric entity level	Boundary
Selection	Geometry geom1: Dimension 0: Boundary 2

Name	Expression	Unit	Description
f	cOx/Gamma_T		Fraction of species in the charge Ox state
Phi_PET	Phi	V	Electrostatic potential at plane of electron transfer (PET)
kf	k0*exp(-alpha*nF_RT*(intop1(Phi) - E0 - Phi_PET))	1/s	Forward rate constant
kb	k0*exp((1 - alpha)*nF_RT*(intop1(Phi) - E0 - Phi_PET))	1/s	Backward rate constant
R_Redox	(-kf*cOx + kb*(Gamma_T - cOx))	$mol/(m^2 \cdot s)$	Rate equation

2.1.2 Functions

Piecewise 1

Function name	Potential
Function type	Piecewise

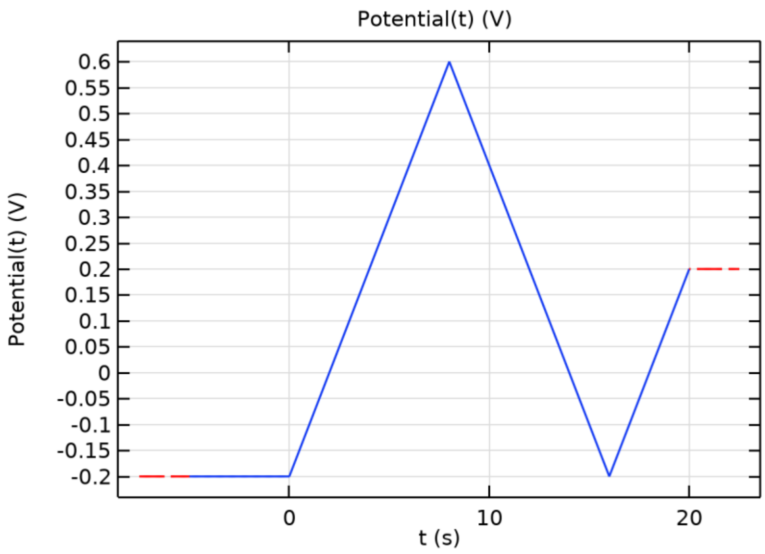

Piecewise 1

DEFINITION

Description	Value
Argument	t
Extrapolation	Constant
Smoothing	No smoothing

DEFINITION

Start	End	Function
-5 [s]	0	OpV_i
0	SegmentDuration	OpV_i+v*t
SegmentDuration	2*SegmentDuration	OpV_sw-v*(t-SegmentDuration)
2*SegmentDuration	2.5*SegmentDuration	OpV_i+v*(t-2*SegmentDuration)

UNITS

Description	Value
Arguments	s
Function	V

2.1.3 Nonlocal Couplings

Integration 1

Coupling type	Integration
Operator name	intop1

SELECTION

Geometric entity level	Boundary
Selection	Geometry geom1: Dimension 0: Boundary 1

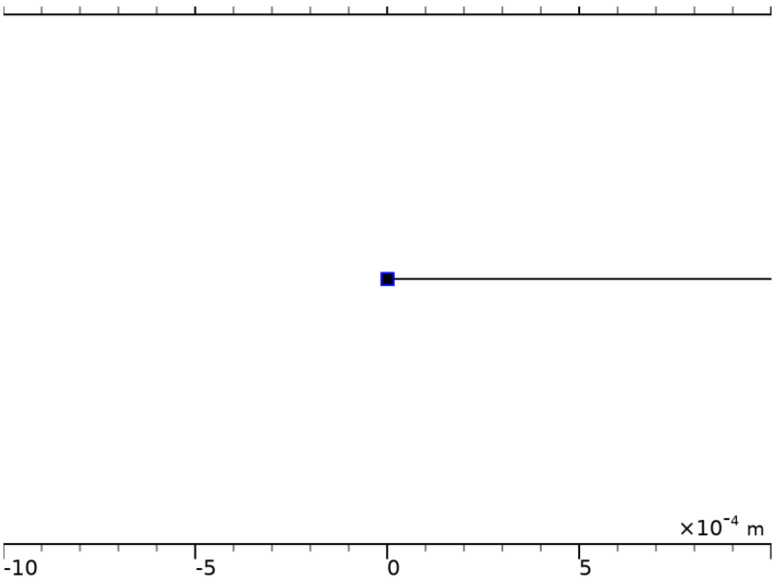

Selection

2.2 GEOMETRY 1

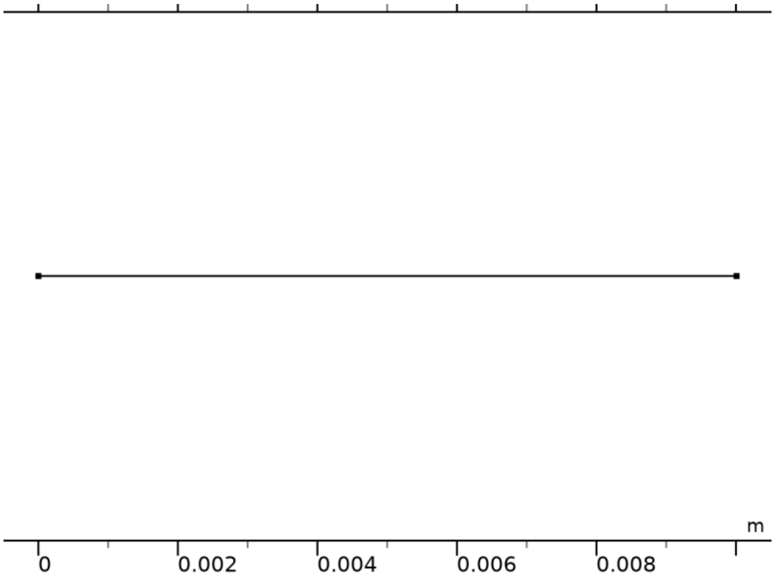

Geometry 1

UNITS

Length unit	m
Angular unit	deg

GEOMETRY STATISTICS

Description	Value
Space dimension	1
Number of domains	2
Number of boundaries	3

2.2.1 Interval 1 (i1)

INTERVAL

Coordinates (m)
0
L

2.2.2 Point 1 (pt1)

POINT

Description	Value
Point coordinate	d1

2.3 TRANSPORT OF DILUTED SPECIES

USED PRODUCTS

COMSOL Multiphysics
Chemical Reaction Engineering Module

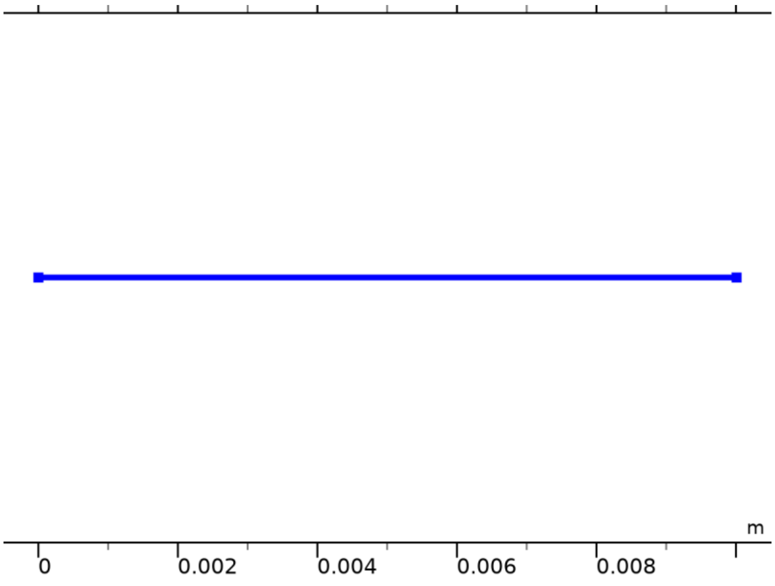

Transport of Diluted Species

SELECTION

Geometric entity level	Domain

Selection	Geometry geom1: Dimension 1: Domain 2

EQUATIONS

$$\frac{\partial c_i}{\partial t} + \nabla \cdot \mathbf{J}_i = R_i$$

$$\mathbf{J}_i = -D_i \nabla c_i - z_i u_{m,i} F c_i \nabla V$$

2.3.1 Interface Settings

Discretization

SETTINGS

Description	Value
Concentration	Linear
Compute boundary fluxes	On
Apply smoothing to boundary fluxes	On
Value type when using splitting of complex variables	Real

Transport Mechanisms

SETTINGS

Description	Value
Convection	Off
Migration in electric field	On
Mass transfer in porous media	Off

Advanced Settings

SETTINGS

Description	Value
Convective term	Nonconservative form

2.3.2 Transport Properties 1

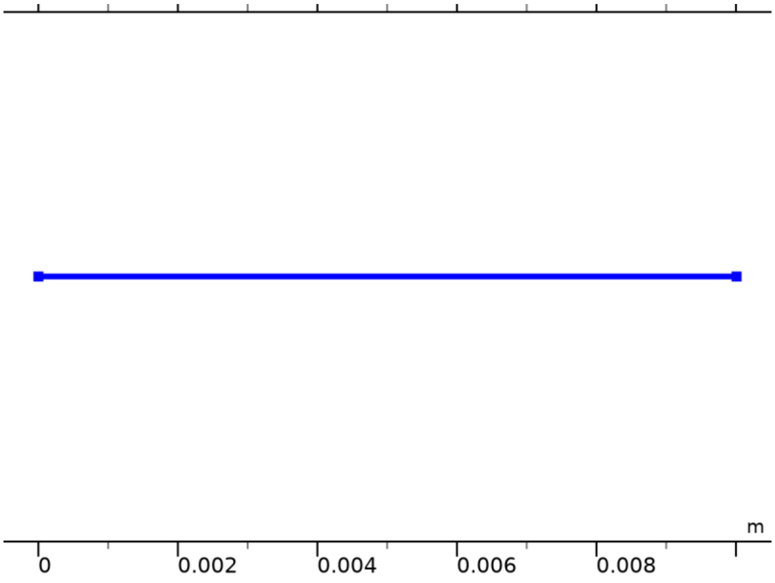

Transport Properties 1

SELECTION

Geometric entity level	Domain
Selection	Geometry geom1: Dimension 1: All domains

EQUATIONS

$$\frac{\partial c_i}{\partial t} + \nabla \cdot \mathbf{J}_i = R_i$$

$$\mathbf{J}_i = -D_i \nabla c_i - z_i u_{m,i} F c_i \nabla V$$

Diffusion

SETTINGS

Description	Value
Source	Material
Material	None
Diffusion coefficient	User defined
Diffusion coefficient	{{D_Cation, 0, 0}, {0, D_Cation, 0}, {0, 0, D_Cation}}
Diffusion coefficient	User defined
Diffusion coefficient	{{D_Anion, 0, 0}, {0, D_Anion, 0}, {0, 0, D_Anion}}

Migration in Electric Field

SETTINGS

Description	Value

Description	Value
Electric potential	Electric potential (es)
Mobility	Nernst - Einstein relation
Charge number	{root.z_Cation, root.z_Anion}

Coordinate System Selection

SETTINGS

Description	Value
Coordinate system	Global coordinate system

Model Input

SETTINGS

Description	Value
Temperature	User defined
Temperature	T

2.3.3 No Flux 1

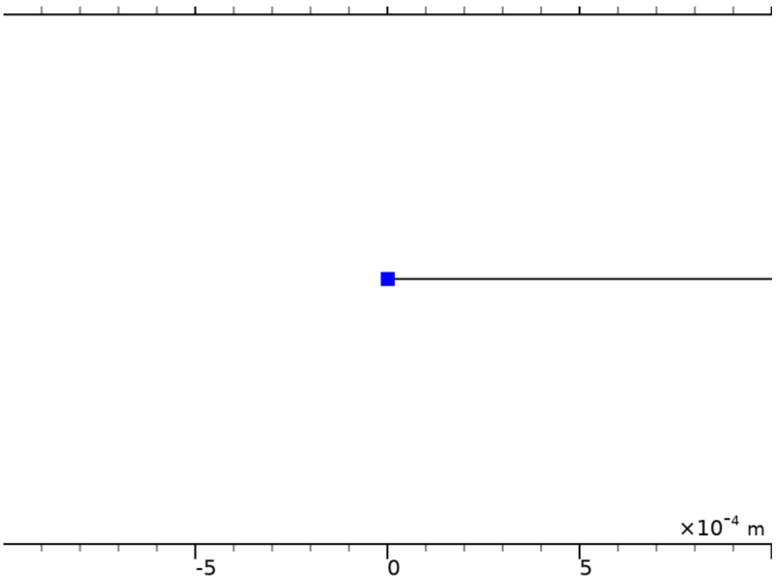

No Flux 1

SELECTION

Geometric entity level	Boundary
Selection	Geometry geom1: Dimension 0: All boundaries

EQUATIONS

$$-\mathbf{n}\cdot\mathbf{J}_i = 0$$

2.3.4 Initial Values 1

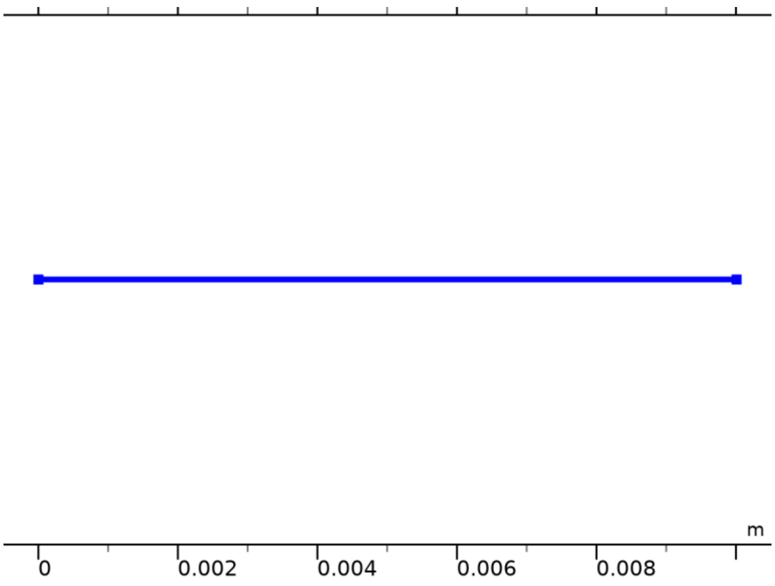

Initial Values 1

SELECTION

Geometric entity level	Domain
Selection	Geometry geom1: Dimension 1: All domains

Initial Values

SETTINGS

**Description**	**Value**
Concentration	{root.cCation_b, root.cAnion_b}

2.3.5 Concentration on BP 4

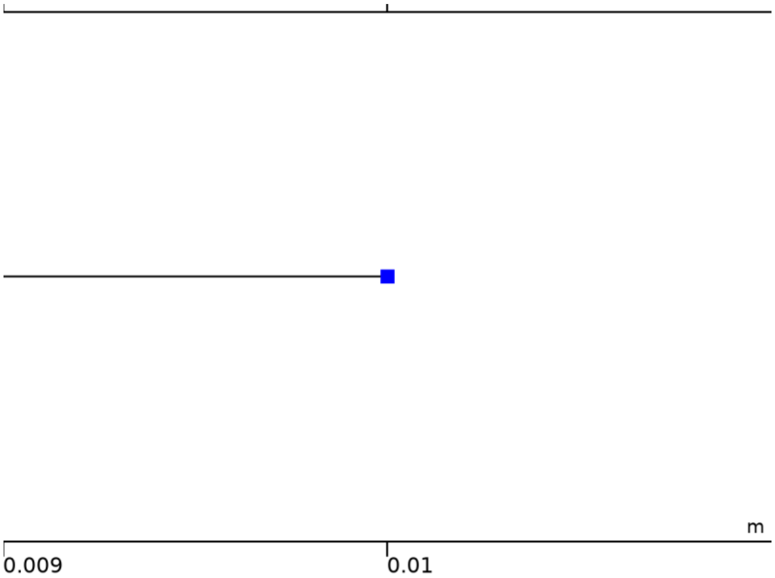

Concentration on BP 4

SELECTION

Geometric entity level	Boundary
Selection	Geometry geom1: Dimension 0: Boundary 3

EQUATIONS

$$c_i = c_{0,j}$$

Concentration

SETTINGS

**Description**	**Value**
Species cCation	On
Species cAnion	On
Concentration	{cCation_b, cAnion_b}

2.4 ELECTROSTATICS

USED PRODUCTS

COMSOL Multiphysics

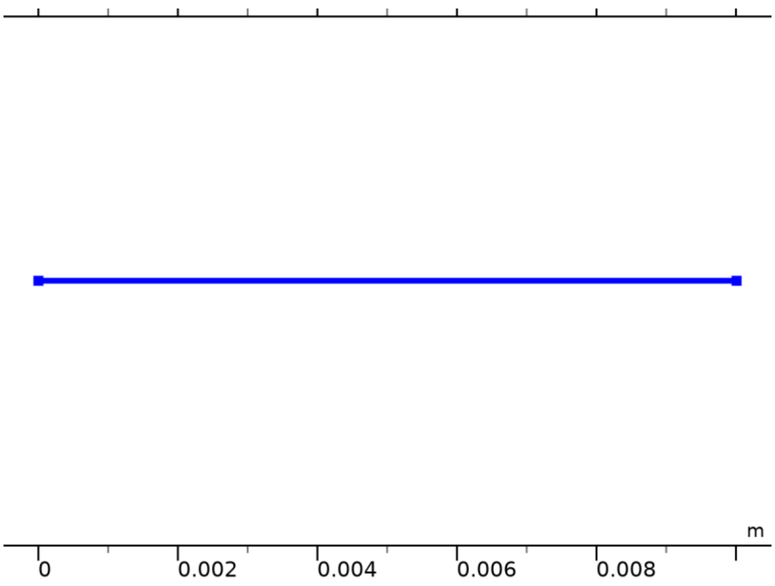

Electrostatics

SELECTION

Geometric entity level	Domain
Selection	Geometry geom1: Dimension 1: All domains

EQUATIONS

$$\nabla \cdot \mathbf{D} = \rho_v$$

$$\mathbf{E} = -\nabla V$$

2.4.1 Interface Settings

Discretization

SETTINGS

**Description**	**Value**
Electric potential	Linear
Value type when using splitting of complex variables	Complex

Manual Terminal Sweep Settings

SETTINGS

**Description**	**Value**
Use manual terminal sweep	Off
Reference impedance	50[ohm]

2.4.2 Charge Conservation - Inner Film

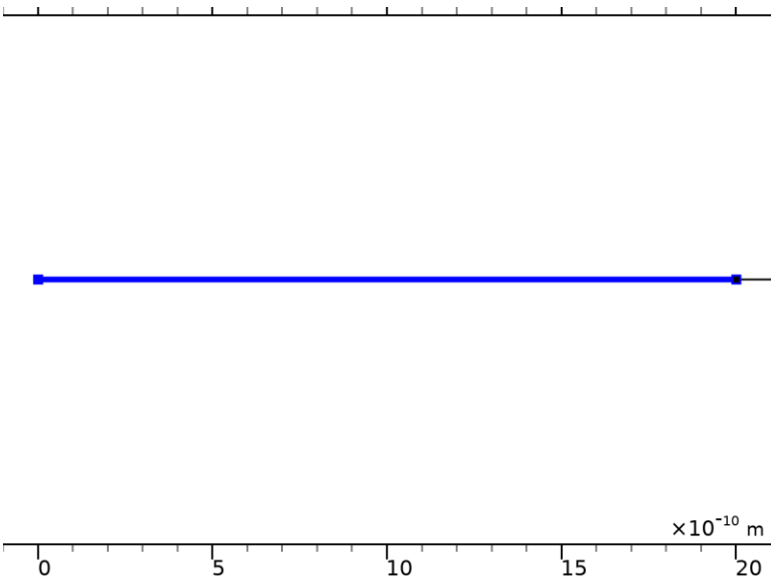

Charge Conservation - Inner Film

SELECTION

Geometric entity level	Domain
Selection	Geometry geom1: Dimension 1: All domains

EQUATIONS

$$\mathbf{E} = -\nabla V$$
$$\nabla \cdot (\epsilon_0 \epsilon_r \mathbf{E}) = \rho_v$$

Constitutive Relation D-E

SETTINGS

Description	Value
Dielectric model	Relative permittivity
Relative permittivity	User defined
Relative permittivity	{{epsilon_1, 0, 0}, {0, epsilon_1, 0}, {0, 0, epsilon_1}}

Coordinate System Selection

SETTINGS

Description	Value
Coordinate system	Global coordinate system

Model Input

SETTINGS

Description	Value
Temperature	User defined
Temperature	T

2.4.3 Zero Charge 1

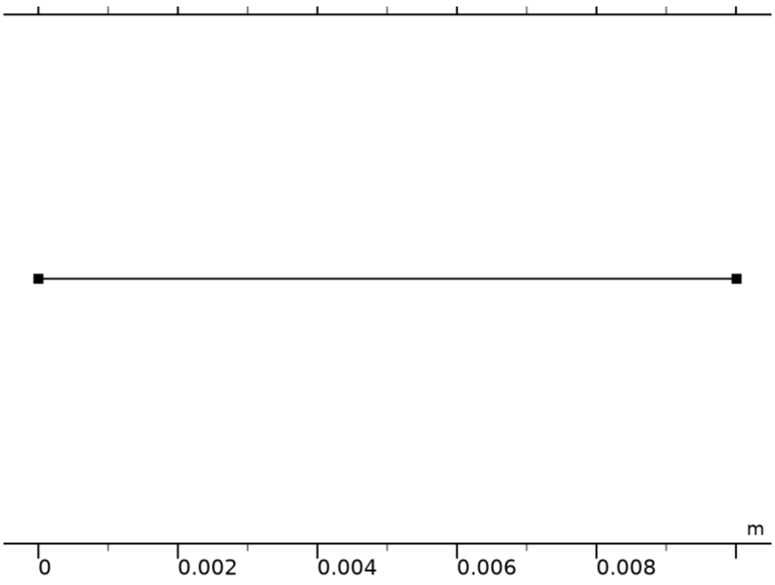

Zero Charge 1

SELECTION

Geometric entity level	Boundary
Selection	Geometry geom1: Dimension 0: All boundaries

EQUATIONS

$$\mathbf{n}\cdot\mathbf{D}=0$$

2.4.4 Initial Values 1

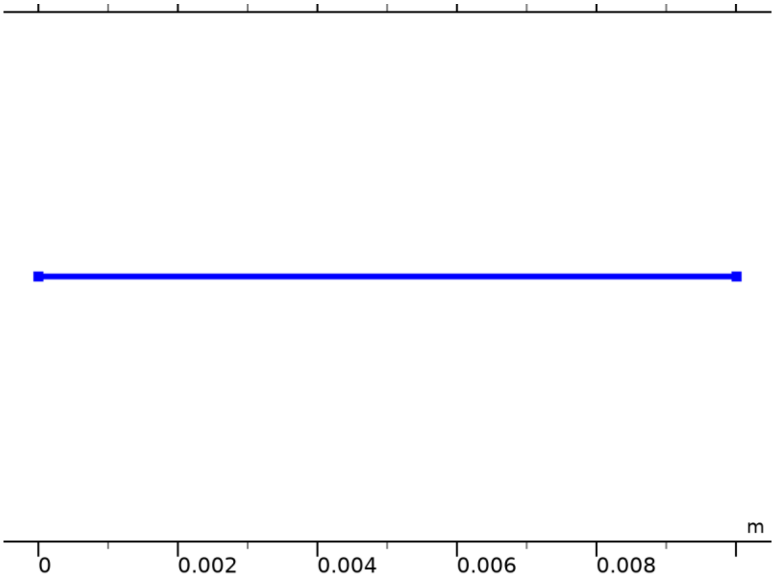

Initial Values 1

SELECTION

Geometric entity level	Domain
Selection	Geometry geom1: Dimension 1: All domains

SETTINGS

Description	Value
Electric potential	0

2.4.5 Charge Conservation - Solution

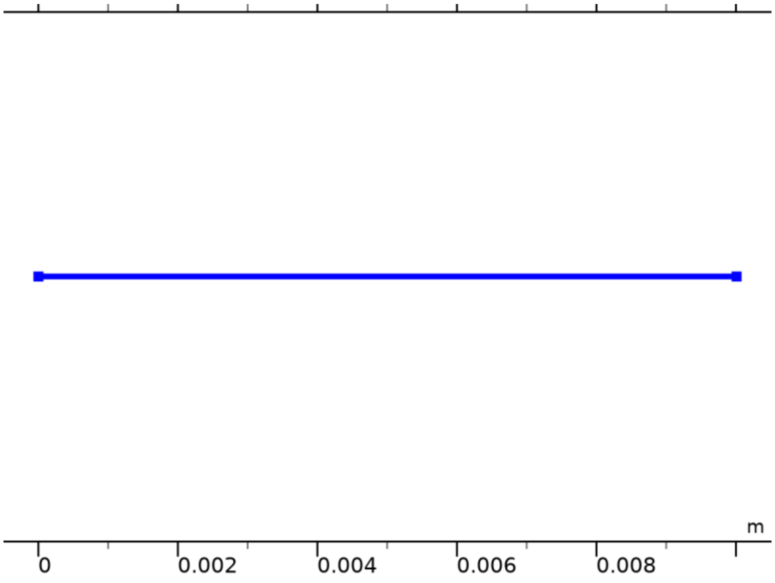

Charge Conservation - Solution

SELECTION

Geometric entity level	Domain
Selection	Geometry geom1: Dimension 1: Domain 2

EQUATIONS

$$\mathbf{E} = -\nabla V$$
$$\nabla \cdot (\epsilon_0 \epsilon_r \mathbf{E}) = \rho_v$$

Constitutive Relation D-E

SETTINGS

Description	Value
Dielectric model	Relative permittivity
Relative permittivity	User defined
Relative permittivity	{{epsilon_s, 0, 0}, {0, epsilon_s, 0}, {0, 0, epsilon_s}}

Coordinate System Selection

SETTINGS

Description	Value
Coordinate system	Global coordinate system

Model Input

SETTINGS

Description	Value
Temperature	User defined
Temperature	T

2.4.6 Ground - Ag|AgCl reference

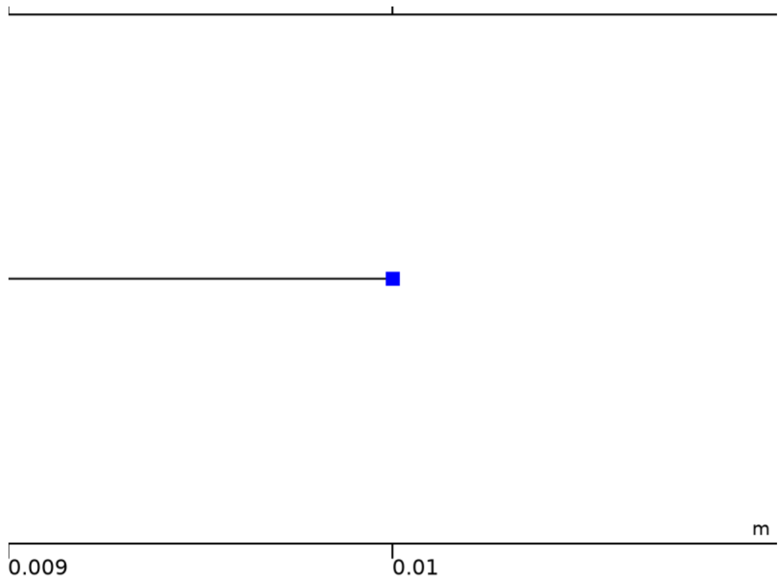

Ground - Ag|AgCl reference

SELECTION

Geometric entity level	Boundary
Selection	Geometry geom1: Dimension 0: Boundary 3

EQUATIONS

$$V = 0$$

Constraint Settings

SETTINGS

Description	Value
Apply reaction terms on	All physics (symmetric)
Use weak constraints	Off
Constraint method	Elemental

2.4.7 PET - Surface Charge Density 1e

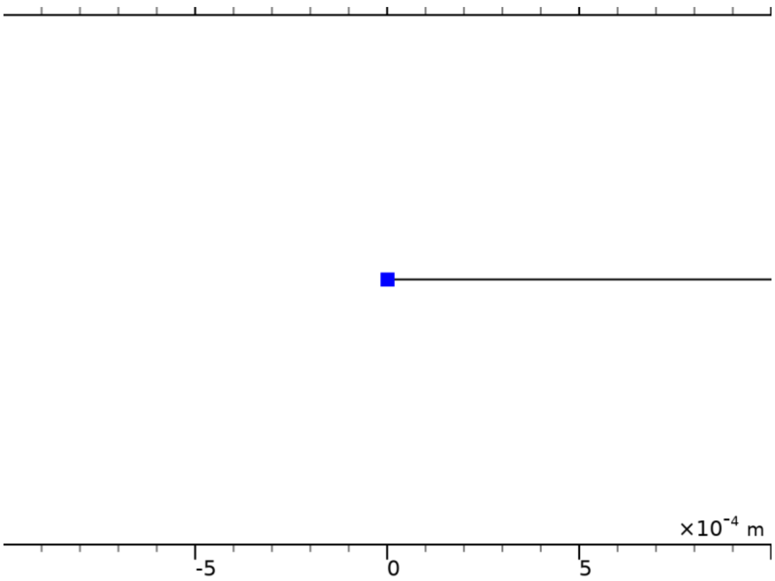

PET - Surface Charge Density 1e

SELECTION

Geometric entity level	Boundary
Selection	Geometry geom1: Dimension 0: Boundary 2

EQUATIONS

$$\mathbf{n} \cdot (\mathbf{D}_1 - \mathbf{D}_2) = \rho_s$$

Surface Charge Density

SETTINGS

Description	Value
Surface charge density	F_const*((zOx*cOx) + (zRed*(cRed)))

Coordinate System Selection

SETTINGS

Description	Value
Coordinate system	Global coordinate system

2.4.8 Electric Potential - Time Dependent

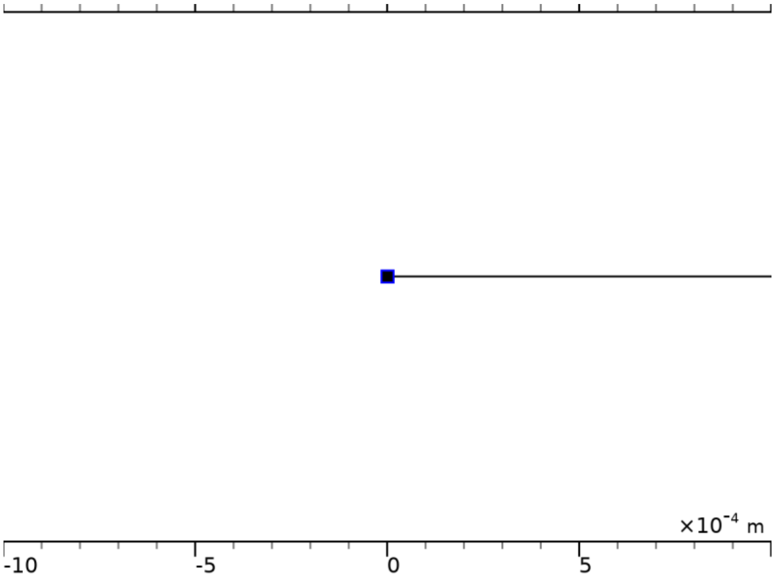

Electric Potential - Time Dependent

SELECTION

Geometric entity level	Boundary
Selection	Geometry geom1: Dimension 0: Boundary 1

EQUATIONS

$$V = V_0$$

Electric Potential

SETTINGS

Description	Value
Electric potential	Potential(t)

2.4.9 Space Charge Density - Multiphysics Coupling

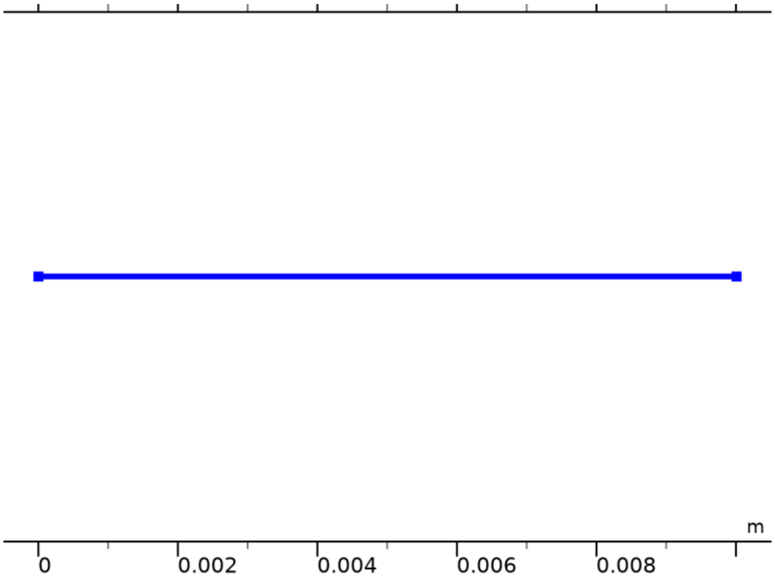

Space Charge Density - Multiphysics Coupling

SELECTION

Geometric entity level	Domain
Selection	Geometry geom1: Dimension 1: Domain 2

EQUATIONS

$$\nabla \cdot \mathbf{D} = \rho_v$$

Coordinate System Selection

SETTINGS

Description	Value
Coordinate system	Global coordinate system

2.5 SURFACE REACTION ON PET

USED PRODUCTS

COMSOL Multiphysics
Chemical Reaction Engineering Module

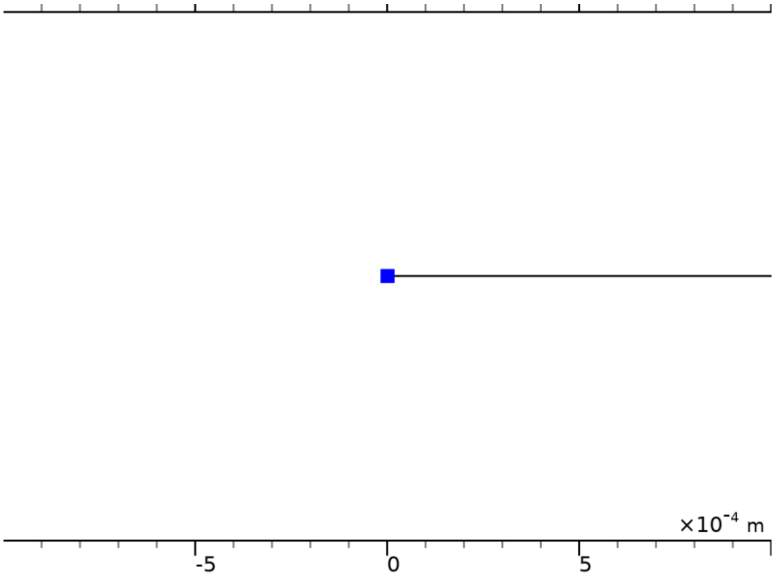

Surface Reaction on PET

SELECTION

Geometric entity level	Boundary
Selection	Geometry geom1: Dimension 0: Boundary 2

EQUATIONS

$$\frac{\partial c_{s,j}}{\partial t} = R_{s,j}$$

$$\theta_i = \frac{c_{s,j}\sigma_i}{\Gamma_s}$$

$$\frac{\partial c_{b,j}}{\partial t} = R_{b,j}$$

2.5.1 Interface Settings

Discretization

SETTINGS

Description	Value
Surface concentration	Linear
Bulk concentration	Linear

2.5.2 Surface Properties 1

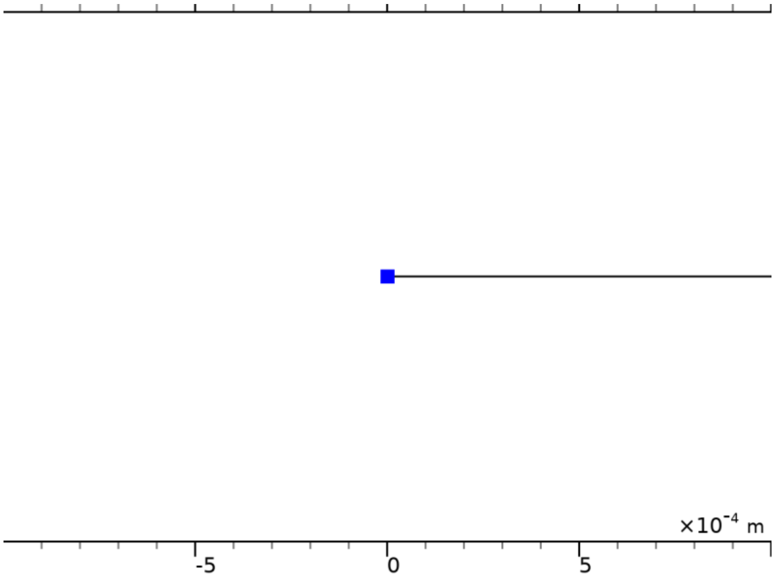

Surface Properties 1

SELECTION

Geometric entity level	Boundary
Selection	Geometry geom1: Dimension 0: All boundaries

EQUATIONS

$$\frac{\partial c_{s,j}}{\partial t} = R_{s,j}$$

$$\theta_i = \frac{c_{s,j}\sigma_i}{\Gamma_s}$$

Sites

SETTINGS

Description	Value
Density of sites	root.Gamma_T
Site occupancy number	{1, 1}

2.5.3 Initial Values 1

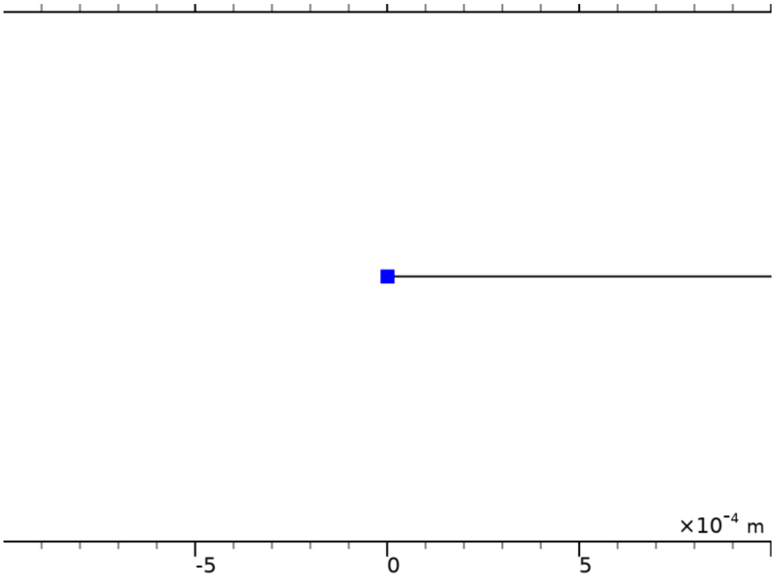

Initial Values 1

SELECTION

Geometric entity level	Boundary
Selection	Geometry geom1: Dimension 0: All boundaries

Initial Values

SETTINGS

Description	Value
Surface concentration	{0, Gamma_T}

2.5.4 Reactions 1

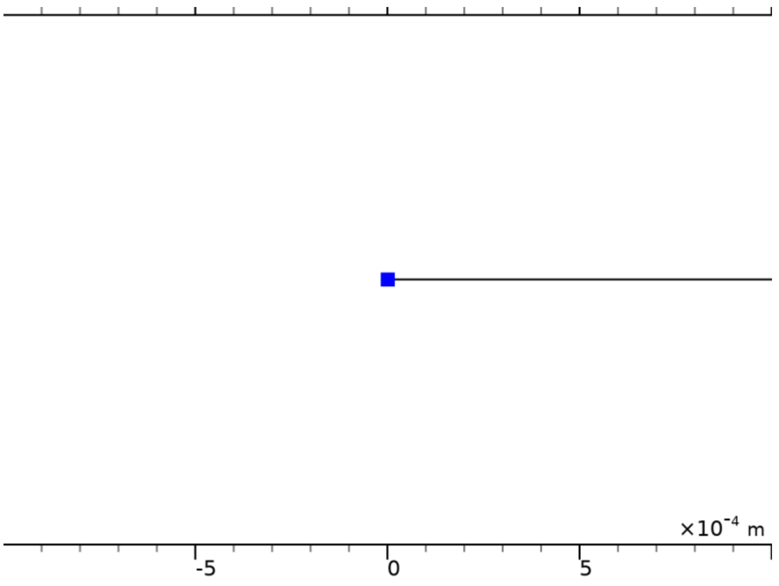

Reactions 1

SELECTION

Geometric entity level	Boundary
Selection	Geometry geom1: Dimension 0: Boundary 2

EQUATIONS

$$\frac{\partial c_{s,i}}{\partial t} = R_{s,i}$$

Reaction Rate for Surface Species

SETTINGS

**Description**	**Value**
Reaction rate for surface species	User defined
Reaction rate for surface species	R_Redox
Reaction rate for surface species	User defined
Reaction rate for surface species	-R_Redox

2.6 MESH 1

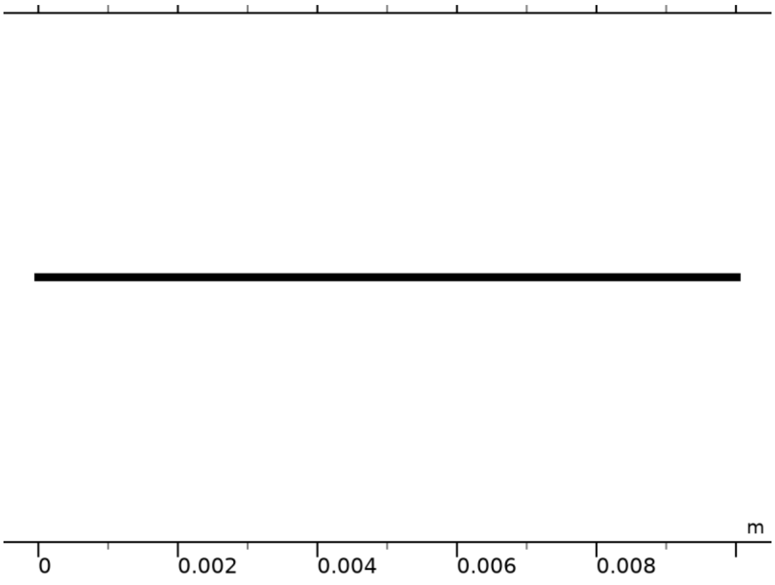

Mesh 1

2.6.1 Size (size)

SETTINGS

Description	Value
Maximum element size	L/100
Minimum element size	3.0E-8
Curvature factor	0.3
Maximum element growth rate	1.3
Custom element size	Custom

2.6.2 Size 1 (size1)

SELECTION

Geometric entity level	Boundary
Selection	Geometry geom1: Dimension 0: Boundary 2

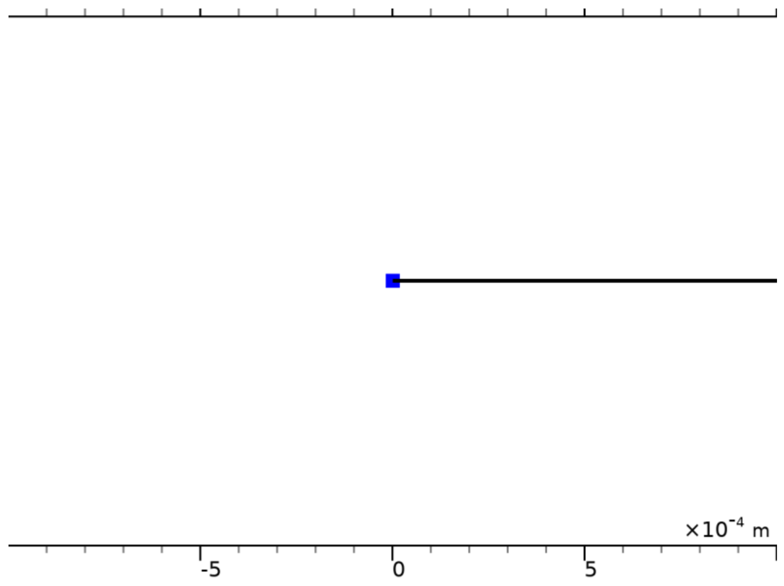

Size 1

SETTINGS

Description	Value
Maximum element size	DebyeLength/20
Minimum element size	3.0E-8
Minimum element size	Off
Curvature factor	0.3
Curvature factor	Off
Resolution of narrow regions	Off
Maximum element growth rate	1.05
Custom element size	Custom

2.6.3 Size 2 (size2)

SELECTION

Geometric entity level	Domain
Selection	Geometry geom1: Dimension 1: Domain 1

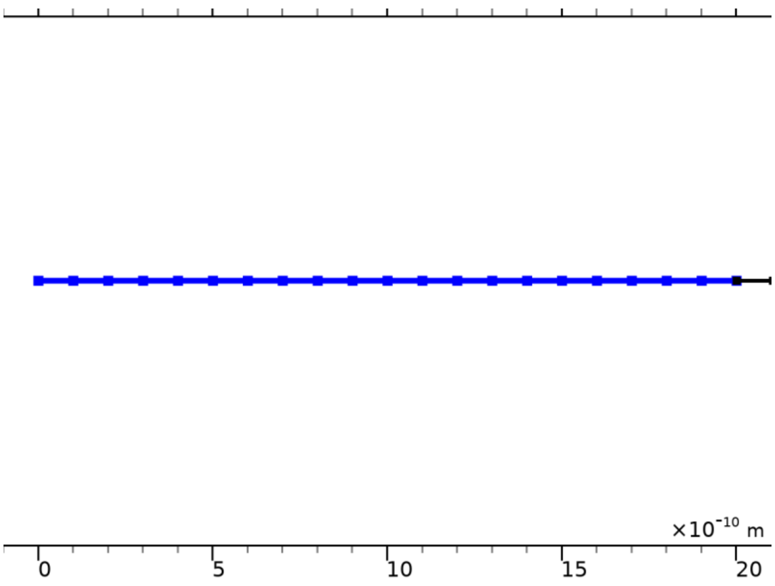

Size 2

SETTINGS

Description	Value
Maximum element size	0.1 [nm]
Minimum element size	3.0E-8
Minimum element size	Off
Curvature factor	0.3
Curvature factor	Off
Resolution of narrow regions	Off
Maximum element growth rate	1.05
Custom element size	Custom

2.6.4 Edge 1 (edg1)

SELECTION

Geometric entity level	Domain
Selection	Geometry geom1: Dimension 1: Domains 1–2

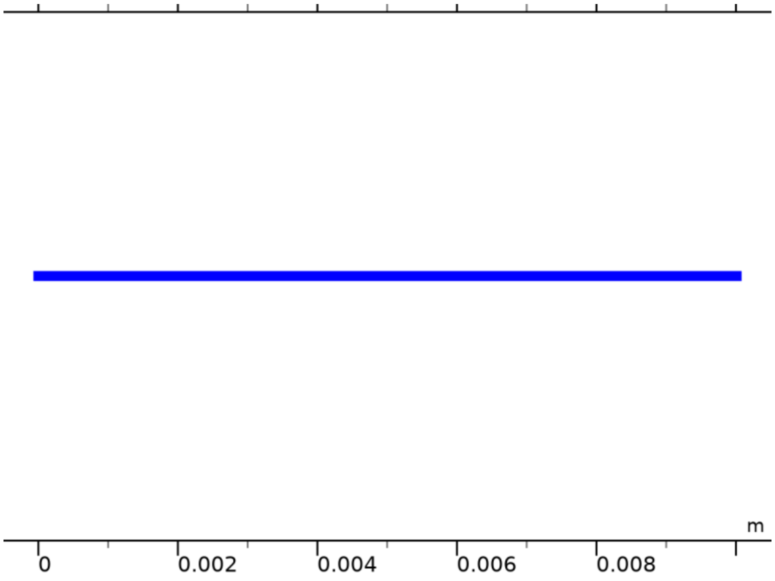

Edge 1

3 Study 1

COMPUTATION INFORMATION

Computation time	1 min 34 s

3.1 PARAMETRIC SWEEP

Parameter name	Parameter value list	Parameter unit
cHb	1, 0.1, 0.01	mol/dm^3

STUDY SETTINGS

Description	Value
Sweep type	Specified combinations
Parameter name	cHb
Unit	mol/dm^3

PARAMETERS

Parameter name	Parameter value list	Parameter unit
cHb	1, 0.1, 0.01	mol/dm^3

3.2 TIME DEPENDENT

Times	Unit
-5, range(0, sampling_time, 2.5*SegmentDuration)	s

STUDY SETTINGS

Description	Value
Include geometric nonlinearity	Off

ADAPTATION

Description	Value
Adaptive mesh refinement	On
Adaptation in geometry	Geometry 1

PHYSICS AND VARIABLES SELECTION

Physics interface	Discretization
Transport of Diluted Species (tds)	physics
Electrostatics (es)	physics
Surface Reaction on PET -1e (sr1e)	physics
Surface Reaction on PET - 2e (sr2e)	physics

MESH SELECTION

Geometry	Mesh
Geometry 1 (geom1)	mesh1

3.3 SOLVER CONFIGURATIONS

3.3.1 Solution 1

Compile Equations: Time Dependent (st1)

STUDY AND STEP

Description	Value
Use study	Study 1
Use study step	Time Dependent

Dependent Variables 1 (v1)

GENERAL

Description	Value
Defined by study step	Time Dependent

RESIDUAL SCALING

Description	Value
Method	Manual

INITIAL VALUE CALCULATION CONSTANTS

Constant name	Initial value source
t	-5, range(0, sampling_time, 2.5*SegmentDuration)
timestep	0.025[s]

Concentration (comp1.cAnion) (comp1_cAnion)

GENERAL

Description	Value
Field components	comp1.cAnion
Internal variables	{comp1.uflux.cAnion, comp1.dflux.cAnion, comp1.tds.dt2Inv_cAnion}

Concentration (comp1.cCation) (comp1_cCation)

GENERAL

Description	Value
Field components	comp1.cCation
Internal variables	{comp1.uflux.cCation, comp1.dflux.cCation, comp1.tds.dt2Inv_cCation}

Electric potential (comp1.Phi) (comp1_Phi)

GENERAL

Description	Value
Field components	comp1.Phi

Surface concentration (comp1.cOx) (comp1_cOx)

GENERAL

Description	Value
Field components	comp1.cOx

Surface concentration (comp1.cRed) (comp1_cRed)

GENERAL

Description	Value
Field components	comp1.cRed

3.3.2 Parametric Solutions 1

cHb=1 (su1)

GENERAL

Description	Value
Solution	cHb=1

cHb=0.1 (su2)

GENERAL

Description	Value
Solution	cHb=0.1

cHb=0.01 (su3)

GENERAL

Description	Value
Solution	cHb=0.01

4 Results

4.1 DATASETS

4.1.1 Study 1/Solution 1

SOLUTION

Description	Value
Solution	Solution 1
Component	Component 1 (comp1)

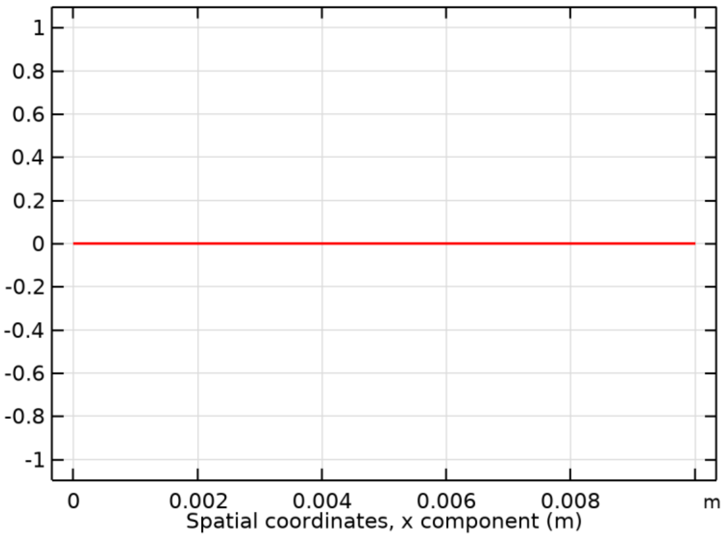

Dataset: Study 1/Solution 1

4.1.2 Study 1/Parametric Solutions 1

SOLUTION

Description	Value
Solution	Parametric Solutions 1
Component	Component 1 (comp1)

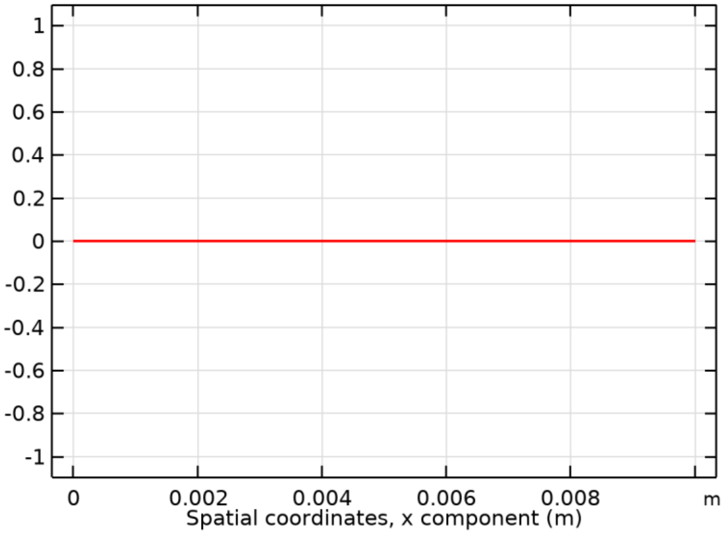

Dataset: Study 1/Parametric Solutions 1

4.1.3 Study 1/Refined Mesh Solution 1

SOLUTION

Description	Value
Solution	Refined Mesh Solution 1
Component	Component 1 (comp1)

4.2 PLOT GROUPS

4.2.1 Current Density

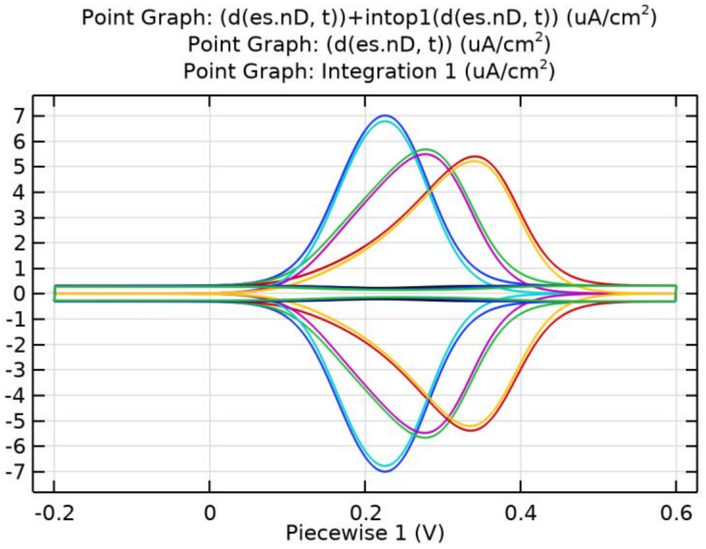

Point Graph: (d(es.nD, t))+intop1(d(es.nD, t)) (uA/cm²) Point Graph: (d(es.nD, t)) (uA/cm²) Point Graph: Integration 1 (uA/cm²)

4.2.2 Surface Species Concentration (sr)

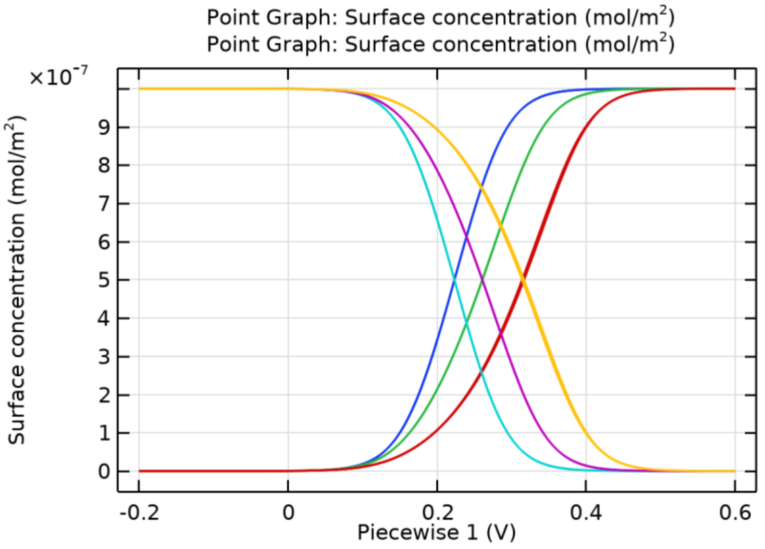

Point Graph: Surface concentration (mol/m²) Point Graph: Surface concentration (mol/m²)

4.2.3 Concentration Supporting Electrolyte (tds)

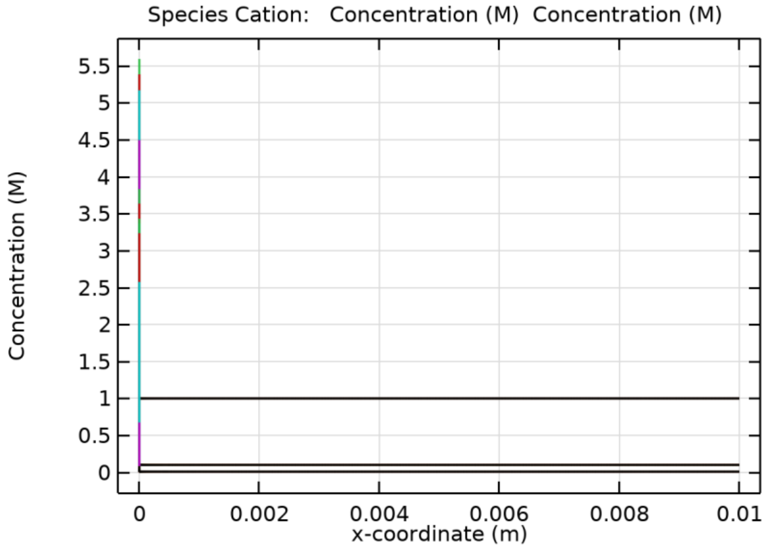

Species Cation: Concentration (M) Concentration (M)

4.2.4 Electric Potential (es)

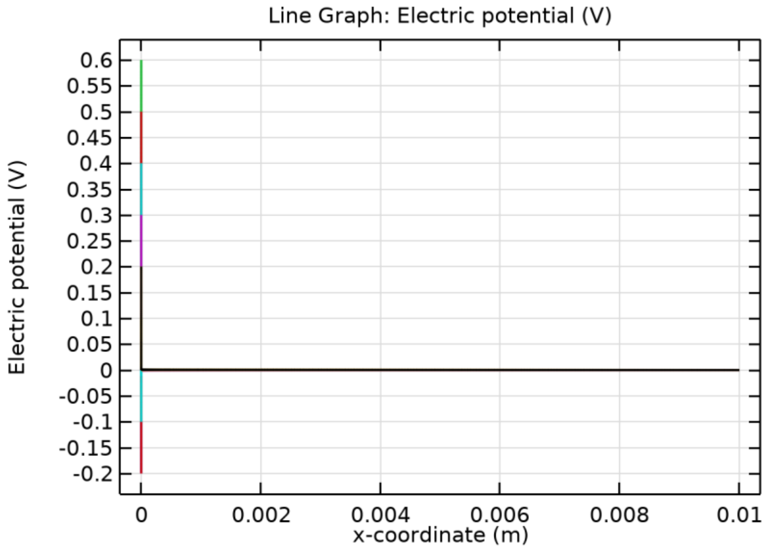

Line Graph: Electric potential (V)
