# Supplementary material for: Finite Element Modeling of the Combined Faradaic and Electrostatic Contributions to the Voltammetric Response of Monolayer Redox Films
Source: Anal Chem. 2022 Sep 7;94(37):12673–82. doi: 10.1021/acs.analchem.2c01976 (PMC9494304; doi:10.1021/acs.analchem.2c01976)
Supplement: Supplementary file 2 — ac2c01976_si_002.pdf [file ac2c01976_si_002.pdf]

Supporting Information

Finite Element Modelling of the Combined Faradaic and Electrostatic Contributions to the Voltammetric Response of Monolayer Redox Films

Katherine J. Levey, [a,b] Martin A. Edwards, [c] Henry S. White,*[d] and Julie V. Macpherson*[a,b]

[a] Department of Chemistry and [b] Centre for Diamond Science and Technology, University of Warwick, Coventry, CV4 7AL, UK

[c] Department of Chemistry & Biochemistry, University of Arkansas, Fayetteville, AR 72701, USA

[d] Department of Chemistry, University of Utah, 315S 1400E, Salt Lake City, UT 84112, USA

*Email- j.macpherson@warwick.ac.uk, white@chemistry.utah.edu

Contents

SI 1. Details of the finite element model implemented in COMSOL Multiphysics................S2

SI 2. The voltammetric response of electrochemically inactive molecular films....................S6

SI 3. Quantification of experimental errors caused by incorrect assumptions on the behavior of the non-faradaic current..S10

SI 4. Simulated voltammetry of electrochemically active molecular films with low surface coverages of redox-active groups S12

SI 5. Calculating the uncompensated resistance of the electrochemical cell.........................S13

SI 6: The R_uC_T transient response at the switching potential S14

SI 7. Influence of supporting electrolyte and scan rate on the voltammetric response S15

SI 8. O^-/R^{2-} and O^+/R^- Redox Monolayers...S18

References……………………………………………………………………………..S21

SI 1. Details of the finite element model implemented in COMSOL Multiphysics

As described in the main text, numerical simulations were used to calculate temporally varying quantities during voltammetry. In this section, we provide additional details on these simulations, which are supported by a COMSOL model report that is supplied as additional supporting information: "The COMSOL-generated report corresponding to the finite element model.PDF."

Quantities calculated in the model are the surface concentrations of the redox species, Γ_i (i = O/R), the distribution of the electric potential, ϕ, and the concentrations of the supporting electrolyte, c_i (i = H^+ or ClO_4^-). The transport and potential in the electrolyte solution are described by the coupled Poisson-Nernst-Planck equations (eqs. 2 and 3 in the main text), where we assume mass transport is by diffusion and migration only (no convective transport). Within the molecular film the electric potential is described by the Laplace equation (eq. 5), as ions cannot penetrate the molecular film. The electron-transfer kinetics are described using Butler Volmer formalization as described by eqs. 9 and 10 in the main text.

Geometry

The equations were solved within the 1D geometry shown in Figure S1. Boundary point 1 represents the metal electrode surface (x = 0) and boundary point 3 the edge of the bulk solution (x = 1 cm). The redox centers are located at the plane of electron transfer (PET; point 2), which is located at $x = d$ (see inset to Figure S1). Region 1-2 represents the film and 2-3 the solution.

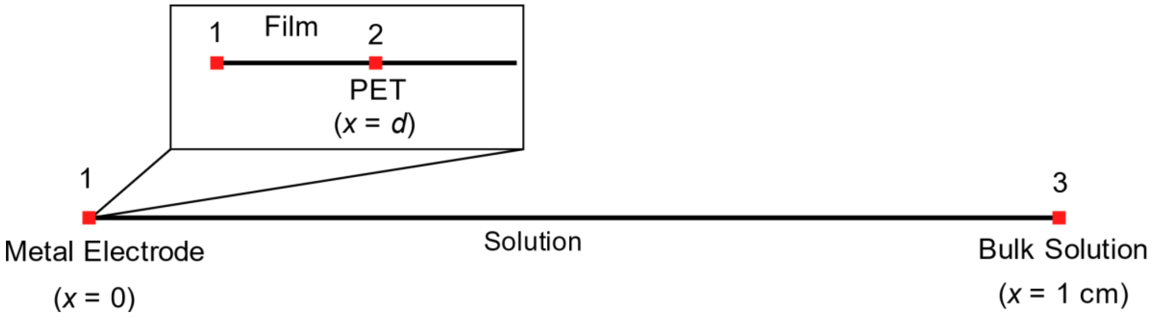

Figure S1. *Representation of the geometry of the domain used in the simulations. Numbered points represent locations where boundary conditions are applied. PET: plane of electron transfer.*

In the simulations, the positions of reference and counter electrodes are located at point 3. The potential of that point is held at 0 V, while the ionic current passing through point 3 is equal to the total current (capacitance and faradaic) at the electrode surface (x = 0).

Potential waveform and initial equilibration

The cyclic voltammetry (CV) waveform is applied using a piecewise linear function, *E*(t), as shown in Figure S2. The model was allowed to equilibrate at the initial potential ($\frac{\partial c_i}{\partial t} = 0; \frac{\partial \Gamma_i}{\partial t} = 0$), for 5 s, between -5 and 0 s, prior to solving the time-dependent equations. By starting the system at equilibrium, the effect of charge flow from the initial formation of the electrical double layer on the voltammetric response is avoided.

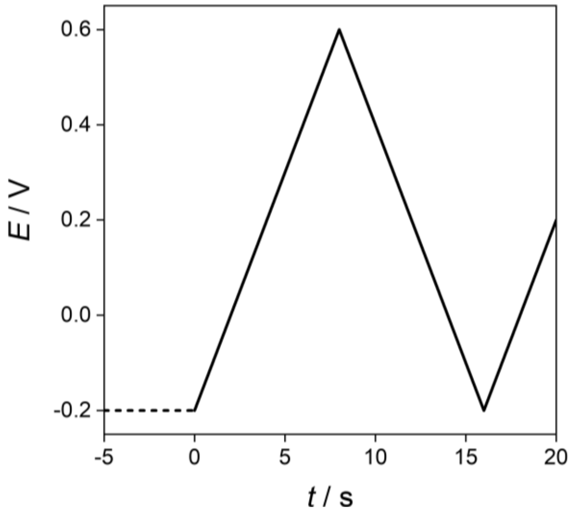

Figure S2. *Example of a piecewise function, E(t), is used to simulate a CV by applying the potential between the metal electrode (boundary point 1; Figure S1) and the bulk solution (boundary point 3). In the example shown, ν = 0.1 V/s and the potential is swept between -0.2 V and +0.6 V.*

Mesh and accuracy

The equations were discretized over a mesh such as that shown in Figure S3, which is finest near the surface of the molecular film (maximum element size on the surface of the film is one twentieth of the Debye length) and coarser in the bulk solution (maximum element size on the is one hundredth of the domain length). The concentration and potential distribution of species were described using linear Lagrange elements. The accuracy of the calculated solutions was confirmed when no appreciable changes were observed in the simulated results when using finer mesh elements or higher solver tolerances. The size of the domain was chosen to be 1 cm, which is a realistic distance between the working and reference/counter electrodes.

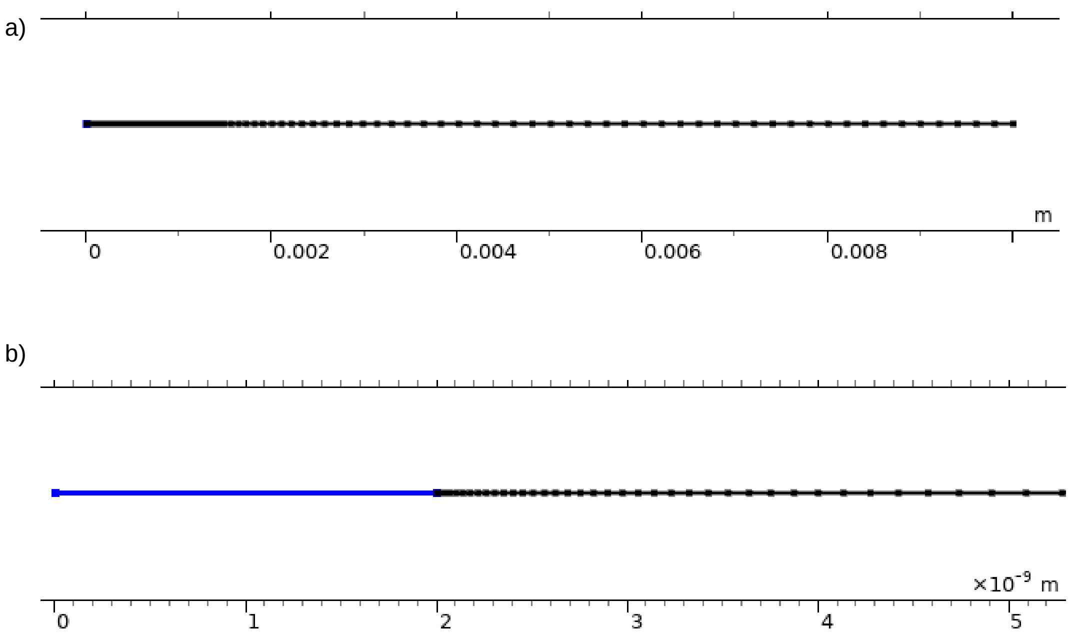

Figure S3. *Example of the mesh used for the simulations for d =2 nm. a) Entire 1 cm wide domain. b) Zoomed-in section of the mesh corresponding to the solution in contact with the molecular film (blue region).*

Current density calculation

The total current density (j) was calculated from the sum of the non-faradaic charging current (j_C) and the faradaic current (j_F). The non-faradaic current density is defined as the rate of change with time of the charge density stored at the metal electrode (boundary point 1) as shown by equation S1, where the "+" sign indicates that the potential gradient at x = 0 is evaluated on the positive side (x > 0) of the interface.

$$j_C = \frac{\partial \sigma_M}{\partial t} = \frac{\partial}{\partial t}\left(\varepsilon_0 \varepsilon_F \left.\frac{\partial \phi}{\partial x}\right|_{x=0^+}\right) \quad (S1)$$

The faradaic current density was calculated from the rate of change of the charge stored at the PET

$$j_F = \frac{\partial \sigma_{PET}}{\partial t} = nF\frac{\partial \Gamma_O}{\partial t} \quad (S2)$$

In each case, the derivatives on the right-hand sides of the equations were calculated through the built-in differentiation operators in COMSOL Multiphysics.

Determination of capacitance

The total current density (C_T) is calculated from the differential definition of capacitance

$$j_C = C_T \frac{dE}{dt} \quad \text{(S3)}$$

by using the observation that the scan rate, v, is the rate of change of potential (i.e., $\frac{dE}{dt} = v$). When rearranged this gives

$$C_T = \frac{j_C}{v} \quad \text{(S4)}$$

Computational software and hardware

Numerical simulations were formulated using COMSOL Multiphysics 5.6™ (COMSOL AB, Sweden) using the Chemical Reaction Engineering Module. They were run on a desktop computer equipped with 64 GB RAM, and took 2 to 5 minutes per voltammogram to run when the simulation was solved in 1 mV potential steps.

SI 2. The voltammetric response of electrochemically inactive molecular films

Influence of treating ions as point charges

As discussed in the main text, the electrolyte ions are treated as point charges when solving the Poisson-Nernst-Planck equations. At high surface charge densities, which occurs at high applied potentials or with the thinnest films, the solution of the equations yields unfeasibly high concentrations of supporting electrolyte ions at the film/electrolyte interface. This can be seen in Figure S4, which plots the simulated electrolyte concentrations obtained when a potential of 0.6 V is applied to the electrochemically inactive film in 1 M $HClO_4$ electrolyte. For the film thickness of $d = 0.1$ nm (Figure S4a), an anion concentration of ~30 M is observed at the film/electrolyte interface, whereas with a film thickness of d = 0.5 nm (Figure S4b), more feasible concentrations, with a maximum value of ~3 M, are observed.

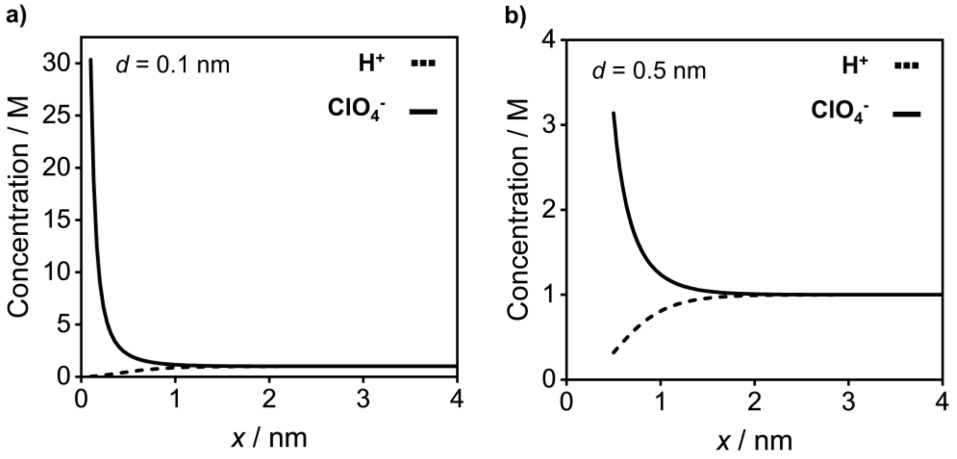

***Figure S4**. Simulated concentration distributions vs distance from the electrode surface for an electrochemically inactive molecular film of thickness a) d = 0.1 nm and b) d = 0.5 nm. Distributions are taken at E = +0.6 V in 1 M of $HClO_4$ supporting electrolyte. All other parameters are as listed in the caption to Figure 3 in the main text. Note the differing concentration scales between a) and b).*

Derivation of analytical solution for the voltammetric response

An analytical expression for the voltammetric response of the electrochemically *inactive* molecular film can be obtained from the Gouy-Chapman-Stern (GCS) model, which assumes that the film and solution behave like two capacitors in series. Figure 2 of the main text shows a plot of the analytical response of an electrochemically inactive film (described below) versus

that from numerical calculations, indicating excellent agreement between the two methods for the conditions chosen (slow scan rate, high solution conductivity).

The analytical expression for the total capacitance density, C_T, was calculated using the equation for computing series capacitance (eq. S5).

$$\frac{1}{C_T} = \frac{1}{C_F} + \frac{1}{C_S} \tag{S5}$$

where the capacitance per unit area (μF/cm^2) of the film (C_F) is assumed to behave like a conventional parallel-plate capacitor and the diffuse layer capacitance density (C_S) is described by Gouy-Chapman theory (for a z:z electrolyte), as given by eqs. S6 and S7, respectively.

$$C_F = \frac{\varepsilon_F \varepsilon_0}{d} \tag{S6}$$

$$C_S = \varepsilon_S \varepsilon_0 \kappa \cosh\left[\frac{z q_e (\phi_{PET} - \phi_S)}{2 k_B T}\right] \tag{S7}$$

$$\kappa = z q_e \left(\frac{2 n^o}{\varepsilon_S \varepsilon_0 k_B T}\right)^{1/2} \tag{S8}$$

Here, ϕ_{PET} is the electric potential at the film/electrolyte interface ($x = d$), k_B is Boltzmann's constant, q_e is the charge of an electron, ε_F and ε_S are the relative dielectric constants of the film and solution, respectively, κ is the reciprocal Debye length (m^{-1}) and n^o is the number concentration of the ions in the electrolyte.[1]

To determine the electric potential at the film surface, ϕ_{PET}, which is an unknown in eq. S7, we use Gauss' law, which states that the overall charge density of the system is zero, as shown by eq. S9.

$$\sigma_M + \sigma_S = 0 \tag{S9}$$

Here, the charge on the metal σ_M is given by

$$\sigma_M = \frac{\varepsilon_F \varepsilon_0}{d} (\phi_M - \phi_{PET}) \tag{S10}$$

and the charge in the solution from the double layer, σ_S

$$\sigma_S = -\varepsilon_S \varepsilon_0 \kappa \frac{2 k_B T}{z q_e} \sinh\left[\frac{z q_e (\phi_{PET} - \phi_S)}{2 k_B T}\right] \tag{S11}$$

The potential at the plane of electron transfer, ϕ_{PET}, for a given applied potential $E = \phi_M - \phi_S$, is obtained by first substituting the expressions S10 and S11 into eq. S9 and setting $\phi_S = 0$ V. The

resulting expression is then numerically solved for the unknown variable, ϕ_{PET}. Having determined the value of ϕ_{PET}, we substitute it into eq. S7 to obtain the solution capacitance density, C_S, which in turn is substituted in eq. S5, along with eq S6, to compute the total interfacial capacitance density, C_T.

Relationship between interfacial potential and capacitance density

The voltammetric response of electrochemically inactive molecular films of varying thicknesses is shown in Figure 2 of the main text. While a minimum in C_T exists at E_{PZC} for all the cases, it is only for the thinnest film that a significant difference in the capacitance is visible over the ±0.6 V scan range.

This response can be understood by considering that C_T consists of two contributions, C_F and C_S (see eq. S5). The former is potential independent, while the latter depends on the potential at the PET (ϕ_{PET} - ϕ_S = ϕ_{PET} - 0 = ϕ_{PET}). With the thicker film and lower C_F, a larger proportion of the applied potential drops over the film (ϕ_M - ϕ_{PET}) and less over the solution. Consequently, the total capacitance changes only a small amount over the entire potential window.

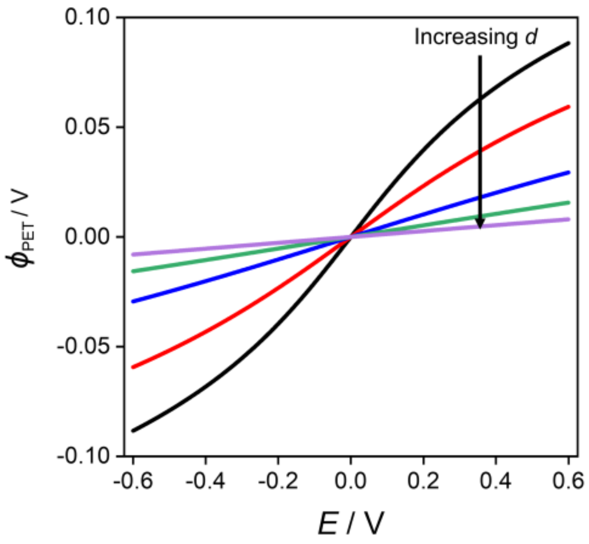

Figure S5. *A plot of simulated ϕ_{PET} as a function of applied potential for electrochemically inactive films of the thickness of 0.1 nm (black), 0.2 nm (red), 0.5 nm (blue), 1 nm (green), 2 nm (purple) in 1 M of supporting electrolyte. All other parameters are as listed in the caption to Figure 2 in the main text.*

This can be seen in Figure S5 which plots the electric potential at the film/electrolyte interface (ϕ_{PET}) vs E for different film thicknesses, corresponding to the curves in Figure 2. For the curve corresponding to the thinnest film (black; d = 0.1 nm), a ~ ± 90 mV potential change is observed

between 0 V and ±0.6 V, corresponding to a change in C_S from a maximum of 660 μF/cm^2 at 0 V to a minimum of 230 μF/cm^2 at ±0.6 V (eq. S7). The resulting ~10% change in C_T from 50 to 55 μF/cm^2 is observable in Figure 2 as a distinct minimum at E = 0 V. In contrast, for the next thickest film d = 0.2 nm (red), the decrease in ϕ_{PET} is only ~± 60 mV between E = 0 V and ±0.6 V, corresponding to a change in C_S from 400 μF/cm^2 to 230 μF/cm^2 over the same potential range. This corresponds to a change in C_T between 30 and 31 μF/cm^2, which is barely distinguishable in Figure 2.

SI 3. Quantification of experimental errors caused by incorrect assumptions on the behavior of the non-faradaic current

In the main text, we showed that the faradaic and non-faradaic components of the voltammetric response of an electrochemically active molecular film were interdependent. The non-faradaic component of the current density showed a decrease during the oxidation/reduction of the film (see Figure 4 in the main text, a portion of which is reproduced below in Figure S6). Moreover, we reported that at potentials away from the redox peak, the non-faradaic component closely resembles that for an electrochemically inactive film (Figure 2, main text). We stated the errors that would ensue if one naïvely assumed the background current of the electrochemically active film to be that of an otherwise identical and electrochemically inert film. Below, we provide details of these calculations.

Figure S6 shows the total and non-faradaic current during voltammetry of two films: a) the main case discussed throughout the paper ($\Gamma_T = 10^{-10}$ mol/cm^2, d = 2 nm, [$HClO_4$] = 1 M), and b) a second plausible scenario ($\Gamma_T = 1\times10^{-12}$ mol/cm^2 and d = 0.75 nm, [$HClO_4$] = 0.1 M) where a significant error could result from incorrect treatment of the non-faradaic current. In both cases, one can clearly see the peak in the total current density (black curve) coinciding with a drop in the non-faradaic current density (solid blue curve). The dashed blue line is a linear interpolation of the non-faradaic contribution to the current density, which is taken from the current values immediately before/after the redox peak. This represents an approximation of the background current anticipated for a non-redox active film. The area between the black and blue curves (total shaded area in grey and blue) represents the charge passed due to faradaic processes, Q_F, which can be related to the total coverage through

$$Q_F = nF\Gamma_T \tag{S12}$$

It is apparent from Figure S6 that using a linear background will result in an underestimation of Q_F equal to the blue shaded area. For the case discussed in the main paper specifically in Figure 4, the area in Figure S6a slightly underestimates the charge, and thus the surface coverage, by ~ 1%. Similar thick films with high surface coverages > 1×10^{-11} mol/cm^2 at high supporting electrolyte concentration > 1 M also produce errors within ~ 1%. In contrast, for the system shown in Figure S6b, corresponding to an electroactive film with d = 0.75 nm, $\Gamma_T = 1\times10^{-12}$ mol/cm^2, in a 0.1 M [$HClO_4$] solution, the area underestimates the charge and, thus, the surface coverage, by ~ 17%. Similarly, if one attempts to measure the (faradaic) peak

current (i_p) from the linear baseline, one obtains a ~15% lower value than from the true value. Simulations for a thinner film with lower surface coverages and at lower supporting electrolyte concentrations lead to larger errors but represent atypical experimental conditions.

Note that while the numbers reported above are for a linear interpolation of the baseline non-faradaic current, from inspection of Figure S6, it is clear that using a higher-order polynomial or another other smooth curve to extrapolate the background current would still result in a considerable amount of area/peak height unaccounted for.

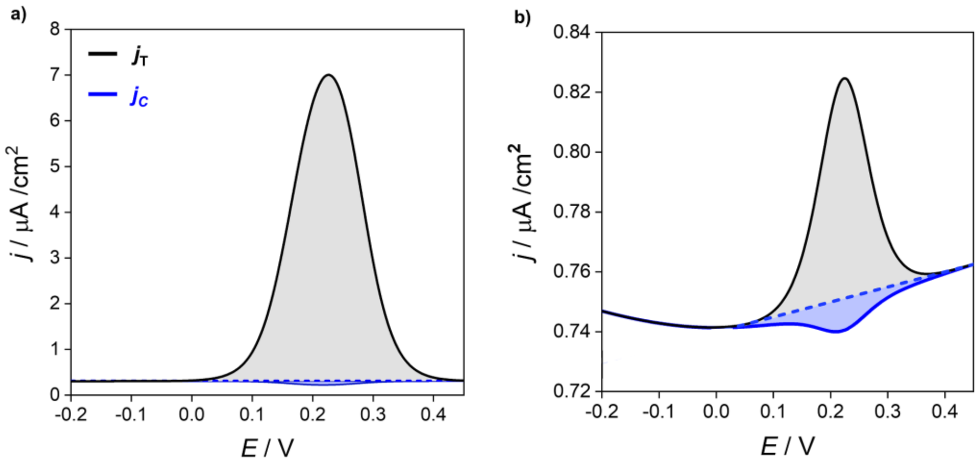

Figure S6. *Current contributions in the voltammetric response of a redox-active (O^+/R) film and assessment of faradaic charge. Curves correspond to the forward scan from a) Figure 4 of the main article ($\Gamma_T = 10^{-10}$ mol/cm^2, d = 2 nm, [$HClO_4$] = 1 M), and b) an example scenario where incorrect treatment could lead to underestimation of faradaic charge ($\Gamma_T = 1\times10^{-12}$ mol/cm^2 and d = 0.75 nm, [$HClO_4$] = 0.1 M). The total shaded area between the curves (when multiplied by v = 0.1 V/s) gives the charge coming from faradaic processes. The dashed line shows a linear interpolation of the non-faradaic response. The grey shaded area indicates the charge that would be assessed using this interpolation as an estimate of the charging current, while the blue shaded area indicates the charge that would be missed if this were used. All other parameters are as listed in the caption for Figure 4.*

SI 4. Simulated voltammetry of electrochemically active molecular films with low surface coverages of redox-active groups

Figure S7 shows voltammetry of an electrochemically active films with a low surface coverage of redox headgroups. These were performed identically to the voltammetry of films with higher coverages shown in Figure 5 of the main text. The expanded current scale in Figure S7 allows the voltammetric peaks to be observed. The *fwhm* calculated from these voltammograms is included in the *fwhm* vs Γ_T plot shown in the inset of Figure 5.

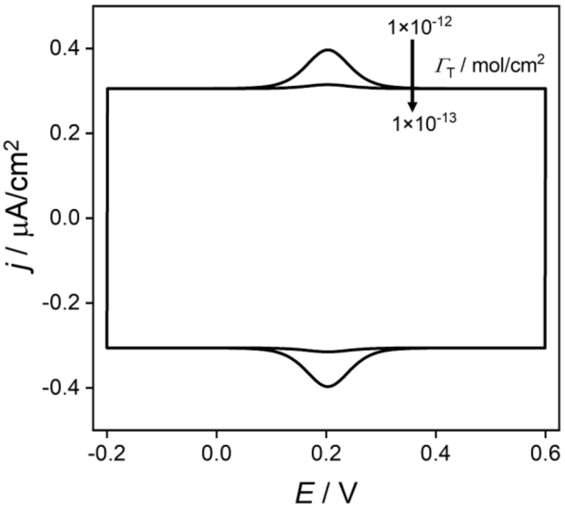

Figure S7. *Simulated voltammetric responses of redox-active (O^+/R) films with surface coverage (Γ_T) of redox groups of 1×10^{-12} and 1×10^{-13} mol/cm^2. The plot corresponds to the data shown in Figure 5. With the exception of the surface coverage of the redox head groups, all other parameters are as listed in the caption to Figure 3.*

SI 5. Calculating the uncompensated resistance of the electrochemical cell

Uncompensated resistance between the working electrode (x = 0 cm) and the reference electrode (x = 1 cm) may impact the voltammetric response and is integral in calculating the double layer charging time. To calculate this resistance, we first determined the conductivity (κ) of the solution:

$$\kappa = F \sum |z_i| u_i c_i \quad (S13)$$

where z_i is the charge, u_i is the mobility and, c_i is the bulk concentration of species i. The mobility of the ions in solution is calculated using the Nernst-Einstein relation:

$$u_i = \frac{|z_i| F D_i}{RT} \quad (S14)$$

Resistance (R_u) is calculated as

$$R_u = \frac{l}{\kappa A} \quad (S15)$$

where A is the electrode area and l is the width of the domain.

For [$HClO_4$] = 1 M, a cell width of l = 1 cm, and using the other physical parameters that go into the model (T = 298.15 K, D_{H+} = 9.3×10^{-5} mol/cm^2, D_{ClO4^-} = 1.8×10^{-5} mol/cm^2) gives R_u = (2.4 × 10^{-4}) Ωm^2/A, which increases to R_u = (2.4 × 10^{-3}) Ωm^2/A and R_u = (2.4 × 10^{-2}) Ωm^2/A for [$HClO_4$] = 0.1 M and 0.01 M, respectively.

SI 6: The R_uC_T transient response at the switching potential

When the direction of the potential scan is switched during voltammetry, the charging current transitions from $\pm \nu C_T$ to $\mp \nu C_T$. At the switching potential, the current changes exponentially with a decay characterized by $\exp(-t/R_uC_T)$, as schematically shown in Figure S8. In our simulations, the asymptotic approach can be seen in voltammograms at high scan rates and/or when the supporting electrolyte concentration is decreased, e.g., in Figure 7a and Figure S9. However, at slower scan rates and high supporting electrolyte concentrations, such as those used in Figures 3 and 4, the rise in current is not visible. This is due to the charging time constant, R_uC_T, being much smaller than the voltammetric timescale, as detailed below.

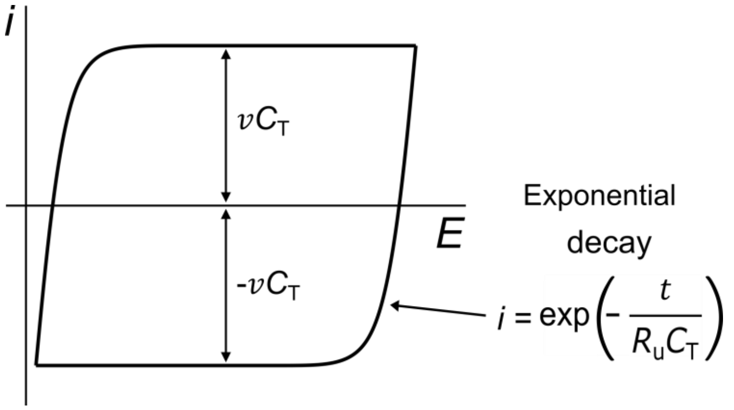

Figure S8. *Schematic of the voltammetric response of a resistor in series with a capacitor.*

For the parameters of the film used in Figures 3-9 (d = 2 nm and ε_F = 7, ε_s = 78, [$HClO_4$] = 1 M), the capacitance density is $C_T = A \times 3.06\ \mu F/cm^2$ (see section S2) at E = 0.6V. The resistance to the electrode in 1 M $HClO_4$ is $R_u = (2.4 \times 10^{-4})\ \Omega m^2 / A$ (see section SI6). These give a time constant of R_uC_T = 7.34 µs. Note, in multiplying these together, we have cancelled out the area term. Similar calculations for [$HClO_4$] = 0.1 M and [$HClO_4$] = 0.01 M give R_uC_T = 71.5 µs and 0.691 ms, respectively.

The exponential nature of the transient current response indicates 95% of the decay will occur in ~$3R_uC_T$. At a scan rate of ν = 0.1 V/s, for the film parameters in the previous paragraph and a solution concentration of [$HClO_4$] = 1 M, this would occur over a potential range of $3R_uC_T \times \nu$ = 2.2 µV. Thus, the response would appear as a vertical line on the plot. In practice, resolving this response would require a careful setup of simulations with appropriately short time steps. In contrast, at the 10 V/s with [$HClO_4$] = 0.01 M, as shown in Figure 8, $3R_uC_T \times \nu$ = 21 mV, and the transient R_uC_T-charging response is visible.

SI 7. Influence of supporting electrolyte and scan rate on the voltammetric response

In Figure 7 of the main article and the related discussion, we described the influence of the supporting electrolyte concentration on the voltammetric response of an electrochemically active molecular film. We reported that larger ohmic drops occur with larger currents (increasing scan rates) and higher solution resistances (lower supporting electrolyte concentrations). The ohmic drop diminishes the interfacial potential that is felt by the redox headgroup and can lead to a distortion of the voltammetric response. For example, an increased peak separation is observed in Figure 7 despite the fast electron transfer kinetics ($k^0 = 10^7$ s^{-1}). For the O^+/R film studied, a positive shift in the average of the anodic and cathodic peak potentials is also observed with the decrease in supporting electrolyte concentration because of the electrostatic interactions. Electron transfer is, therefore, less thermodynamically favorable, and a greater applied potential is required to fully oxidize the film. In this section, we provide voltammograms from which the peak potentials in Figure 7b were determined and take a deeper look at the potential and ion distribution during these simulations.

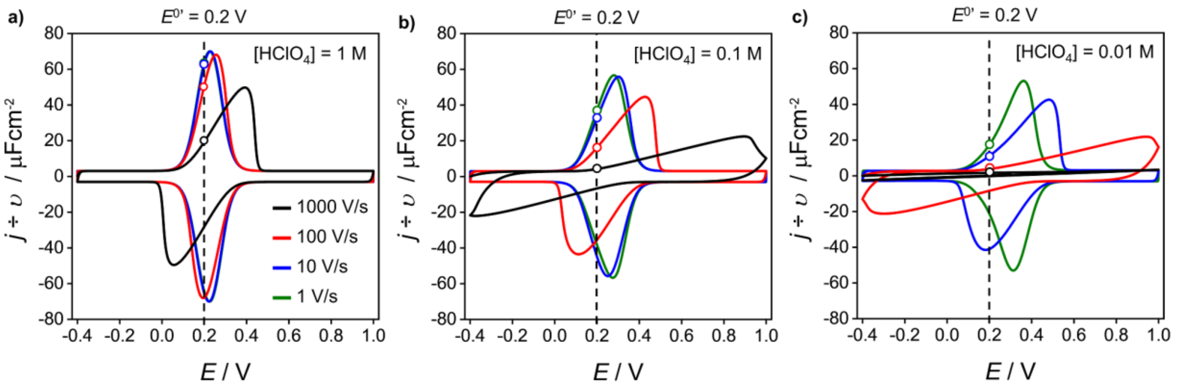

***Figure S9**. Plots of the simulated voltammetric response for a redox-active (O^+/R) film at different supporting electrolyte concentrations of a) 1 M, b) 0.1 M and c) 0.01 M. The voltammograms are shown for scan rates at 1 V/s (green), 10 V/s (blue), 100 V/s (red) and 1000 V/s (black). These voltammograms were used to determine the data shown in Figure 7a. $k^0 = 10^7$ s^{-1}; all other parameters are as listed in the caption to Figure 3.*

Representative voltammograms (1, 10, 100 & 1000 V/s) used to calculate the peak potentials shown in Figure 7b are shown in Figure S9. The current densities are normalized by the scan rate, which allows all voltammograms to be presented on the same scale. The trend of the peak splitting in these voltammograms, as a function of scan rate and supporting electrolyte concentration, can be understood qualitatively by inspection of Figure S10, which shows the i)

electric potential and ii) supporting electrolyte concentration distributions at $E = E^{0'} = +0.2$ V (forward scan). The corresponding points where the distribution plots are extracted are indicated by circles in Figure S9. Ohmic drop ($V = iR_u$) can be seen by the linear potential change across the 1 cm domain (up to the edge of the electrical double layer). It increases with increasing currents (higher scan rates) and/or increasing uncompensated resistance (lower concentration). Ohmic drop causes only a portion of the applied potential to act as a driving force for electron transfer. The driving force is seen in the insets to part i, as the potential drop between the electrode (x = 0 nm; ϕ = 0.2 V) and the plane of electron transfer (x = 2 nm; ϕ various >0 V).

The ohmic drop contribution can also be approximated from the voltammogram and the solution resistance. As an example, the 1000 V/s voltammogram in 1 M electrolyte (black curve in Figure S9a) has a current density of ~2 mA/cm^2 (=1000 V/s × 20 µF/cm^2) at E = 0.2 V. Multiplying by the uncompensated resistance of $R_u = (2.4 \times 10^{-4})\ \Omega m^2 / A$ (section SI6) gives a potential drop of iR_u ~50 mV. This value agrees with the ~ 50 mV drop in potential over the electrolyte shown in the potential profile Figure 10ai (black curve).

In Figure S9c, which shows the lowest supporting electrolyte concentration (0.01 M), the potential profile for the voltammogram at the largest scan rate (black curve, Figure S10c) is inverted across the film, relative to that at slow scan rates (1 – 10 V/s). The supporting electrolyte concentration distributions indicate that the electrode is charged negatively at E = 0.2 V. This can be attributed to the increases in the rise time (discussed in section SI6) associated with electrode charging. The timescale of the electrode charging is slow enough relative to the scan rate that the structure of the double layer cannot keep up with the change in potential.

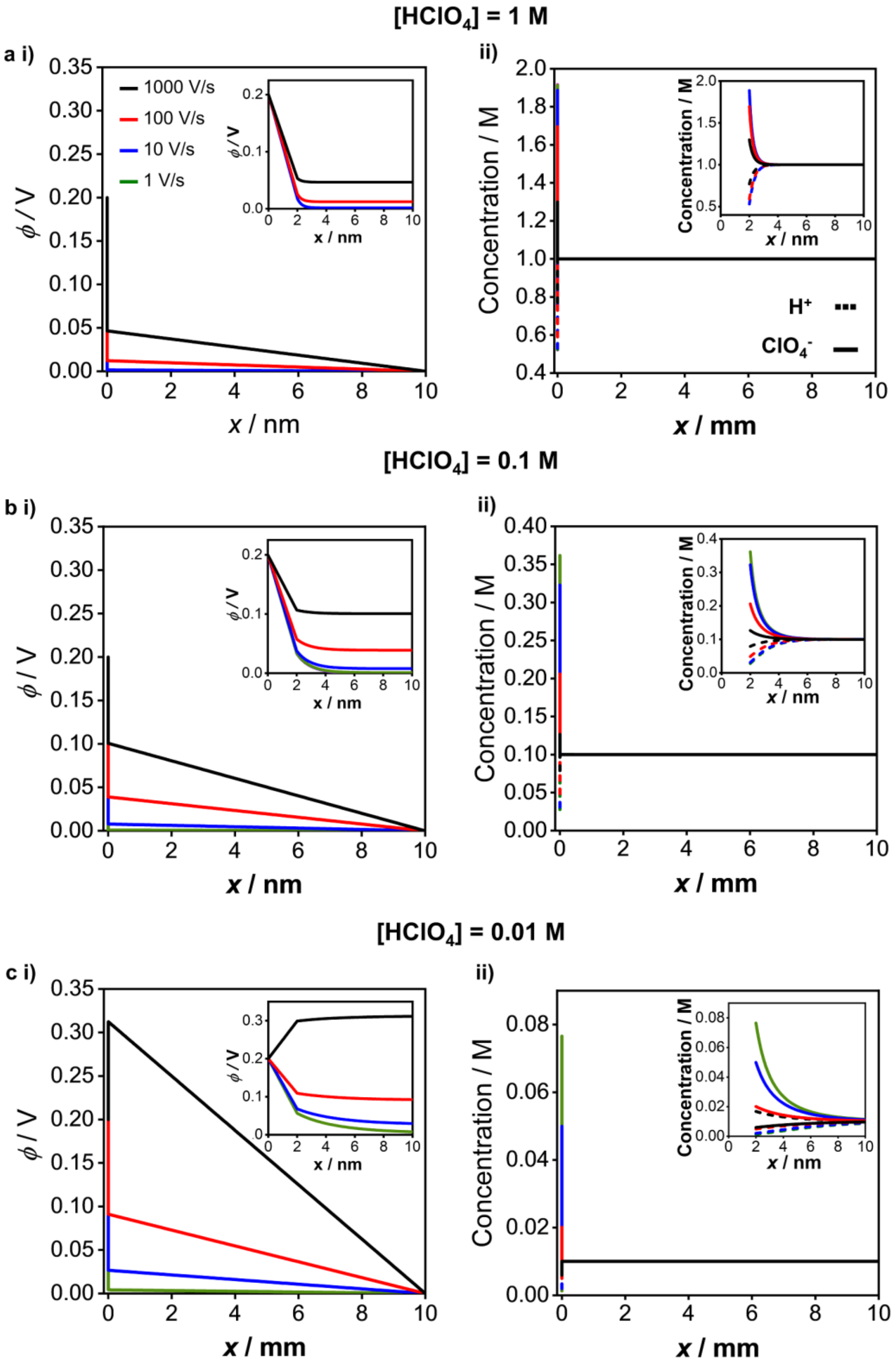

Figure S10. *Plots of the simulated i) potential and ii) electrolyte concentration vs distance from the electrode surface for a redox-active (O^+/R) film at E = +0.2 V (forward scan) for the voltammograms shown in Figure S9. Each pair of plots was obtained at a different supporting electrolyte concentration of a) 1 M, b) 0.1 M and c) 0.01 M and 4 different scan rates (see legend). Note, different concentration scales are used in each plot.*

SI 8. O^-/R^{2-} and O^+/R^- Redox Monolayers

The finite element simulations can readily be extended to redox systems other than the $O^{+/}R$ couple. Figure S11 shows the (i) voltammetric and (ii) interfacial potential profiles of the (a) O^-/R^{2-} (n = 1) and (b) O^+/R^- (n = 2) redox film couples, with all other parameters of the redox film kept as described in Figure 3. The dotted lines in Figure S11a,b (i) represent the nernstian responses.

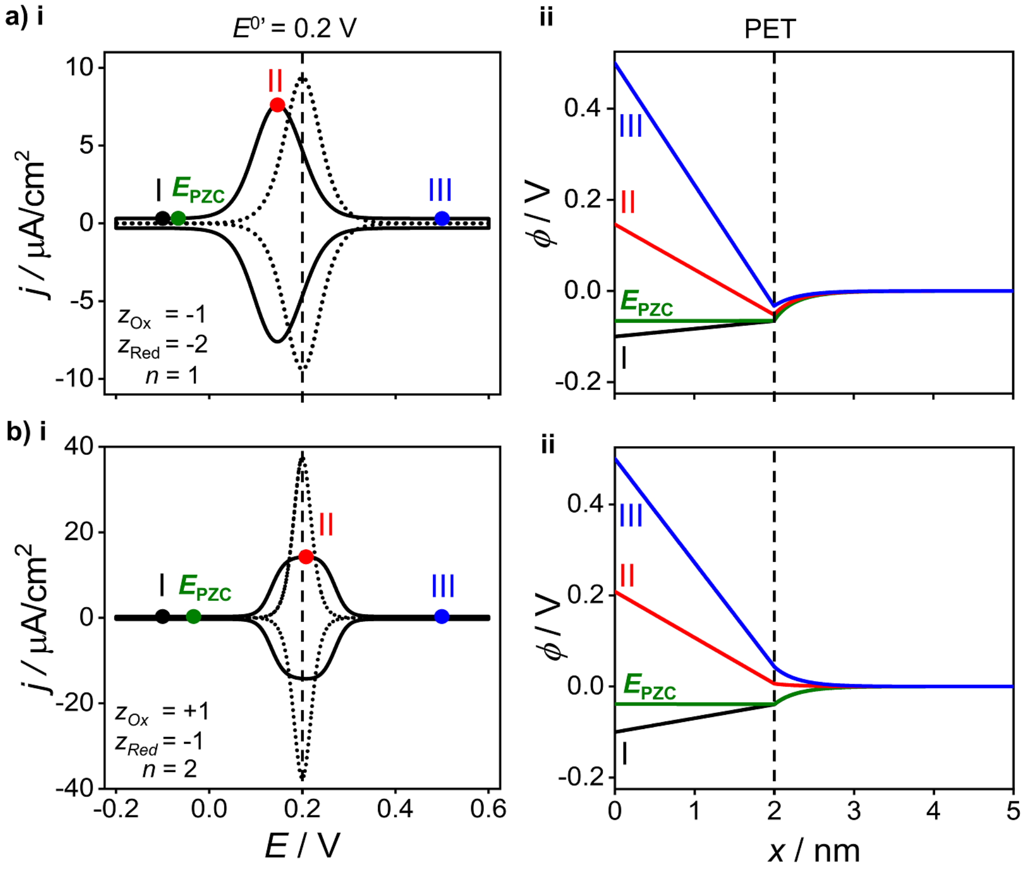

Figure S11. Simulated voltammetric responses (i) and electric potential profiles (ii) for redox-active films with different redox reactions: (a) O^-/R^{2-} and (b) O^+/R^-. Electric potential profiles correspond to labelled points on the voltammograms. Potentials are I (E = -0.100 V), II ($E = E_p$; 0.146 V and 0.208 V for (a) and (b), respectively), III (E = 0.500 V), and E_{pzc} = -0.066 V and -0.039 V, for (a) and (b), respectively. Simulation parameters as in Figure 3.

For the O^-/R^{2-} redox film (Figure S11a), both halves of the redox couple are negatively charged leading to a negative shift of the wave relative to $E^{0'}$ (from 0.200 V to 0.146 V), and a larger *fwhm* (121.0 mV, compared to 90.6 mV for the nernstian case). The negative shift of the wave is due to the potential difference driving electron transfer, ($\phi_M - \phi_{PET}$), now being greater than the applied E. This is a consequence of the inverted potential drop between the PET and bulk

solution ($\phi_{PET} - \phi_S$), as seen in Figure S11aii, which arises due to both forms of the redox system carrying a negative charge. Thus $\sigma_{PET} < 0$, irrespective of the oxidation state of the film. Additionally, unlike the O^+/R case discussed previously where E_{pzc} = 0.000 V, the O^-/R^{2-} monolayer has a E_{pzc} = -0.066 V, corresponding to a potential where the film is in the R^{2-} state. At this potential, the negative charge at the PET is fully compensated for by the excess positive charge in the diffuse layer, and the electric field within the film is 0 as can be seen by the green curve in Figure S11aii.

Figure S11b corresponds to the O^+/R^- redox couple, which is a two-electron transfer process resulting in charge reversal at the PET upon oxidation or reduction of the film. To model this case, the reaction is split into two individual one electron transfer steps,[17] eq. S16 and eq. S17. Here we have introduced a new surface species P^{z+1}, the product of the first oxidation of R^z.

$$R^z \leftrightharpoons P^{z+1} + e \qquad E_1^{0'} \tag{S16}$$

$$P^{z+1} \leftrightharpoons O^{z+2} + e \qquad E_2^{0'} \tag{S17}$$

Each 1e step has its own formal potential, $E_1^{0'}$ and $E_2^{0'}$, and own rate constants (k_f and k_b), which are described by the Butler-Volmer formalism (eqs. 9 and 10 in the main text).

Values of $E_1^{0'}$ and $E_2^{0'}$ are chosen such that $(E_1^{0'} + E_2^{0'})/2$ = 0.2 V, to allow for comparison to the previously discussed redox systems (main text) with $E^{0'}$ = 0.2 V. For the example voltammogram shown in Figure S11bi, the first step ($E_1^{0'}$ = 0.3 V) is less energetically favorable than the second step ($E_2^{0'}$ = 0.1 V), i.e., $E_1^{0'} > E_2^{0'}$. For this parameter choice, the second electron transfer occurs rapidly following the first, which is typical of many two electron processes, and a single broad asymmetric peak is observed, with a maximum current at E = 0.208 V, which is very close to that of the nernstian case.

To further demonstrate the role of the values $E_1^{0'}$ and $E_2^{0'}$ on the voltammetric response, voltammograms were simulated in which $E_1^{0'}$ is fixed at 0.2 V and $E_2^{0'}$ is varied. The results are shown as solid lines in Figure S12. In these simulations, the standard rate constant for each reaction is set at 1×10^7 s^{-1}, which is sufficiently fast to appear reversible on the voltammetric timescale. Plichon and Laviron evaluated the nernstian response of such a system (no electrostatic interactions or background charging capacitance) by assuming that the entire applied potential is dropped across the monolayer and are shown by the dotted line in Figure S12.[2] Here the capacitance contribution in the electrostatic model is barely greater than the width of the line used in the plot.

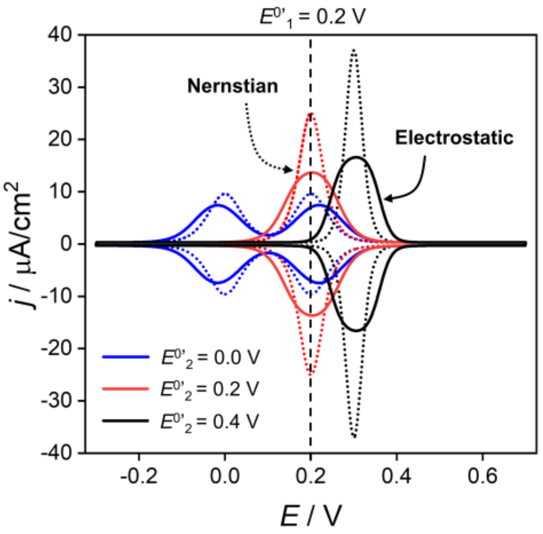

Figure S12. *Simulated voltammetric response of redox-active ($O^+/P^0/R^-$) SAM film with $E_1^{0'}$ = +0.2 V and $E_2^{0'}$ varied (see legend). The voltammetric response is shown when the electrostatic interactions are either excluded (dotted lines) or included (solid lines) in the finite element simulations. The supporting electrolyte concentration is set at 1 M, v = 0.1 V/s and for both reactions $k^0 = 1 \times 10^7$ s^{-1} (reversible). All other parameters are as stated in the caption to Figure 3.*

For all $E_1^{0'}/E_2^{0'}$ combinations, with electrostatic interactions included, lower peak currents and larger *fwhm* result compared to the nernstian description. When $E_2^{0'} \gg E_1^{0'}$ (blue dotted curve; $E_1^{0'}$= 0.2 V, $E_2^{0'}$= 0.4 V) i.e. the second step is less thermodynamically favorable than the second a splitting of the voltammetric response into two waves is seen, as originally described by Plichon and Laviron.[2] When the electrostatic interactions are considered (solid blue lines), each peak is shifted away from the two peaks as described above for a 1e transfer system. In contrast, when $E_2^{0'} \ll E_1^{0'}$ a single peak is observed. Each of the three voltammetric responses for the O^+/R^- 2e redox system are centered on the average of $E_1^{0'}$ and $E_2^{0'}$ as described by Plichon and Laviron.[2] The case shown by the black curve is the most similar to the voltammetric response plotted in Figure S11b, however, the simulations for Figure S11b used $E_1^{0'}$ = 0.3 V and $E_2^{0'}$ = 0.1 V so that the average of the two $E^{0'}$s matched the formal potential of the other case discussed in the figure.

References

(1) Smith, C. P.; White, H. S. Theory of the Interfacial Potential Distribution and Reversible Voltammetric Response of Electrodes Coated with Electroactive Molecular Films. *Anal. Chem.* **1992**, *64* (20), 2398–2405. https://doi.org/10.1021/ac00044a017.

(2) Plichon, V.; Laviron, E. Theoretical Study of a Two-Step Reversible Electrochemical Reaction Associated with Irreversible Chemical Reactions in Thin Layer Linear Potential Sweep Voltammetry. *J. Electroanal. Chem.* **1976**, *71* (2), 143–156. https://doi.org/10.1016/S0022-0728(76)80030-7.
